# Supplementary material for: In Vivo Delivery of a DNA-Encoded Monoclonal Antibody Protects Non-human Primates against Zika Virus
Source: Mol Ther. 2019 Apr 5;27(5):974–85. doi: 10.1016/j.ymthe.2019.03.005 (PMC6520333; doi:10.1016/j.ymthe.2019.03.005)
Supplement: Document S1. Figures S1–S8 and Tables S1 and S2 [file mmc1.pdf]

YMTHE, Volume *27*

Supplemental Information

In Vivo Delivery of a DNA-Encoded Monoclonal Antibody Protects Non-human Primates against Zika Virus

Rianne N. Esquivel, Ami Patel, Sagar B. Kudchodkar, Daniel H. Park, Karin Stettler, Martina Beltramello, Jeffrey W. Allen, Janess Mendoza, Stephanie Ramos, Hyeree Choi, Piyush Borole, Kanika Asija, Mamadou Bah, Shareef Shaheen, Jing Chen, Jian Yan, Amy C. Durham, Trevor R.F. Smith, Kate Broderick, Ghiabe Guibinga, Kar Muthumani, Davide Corti, Laurent Humeau, and David B. Weiner

Supplemental Figures

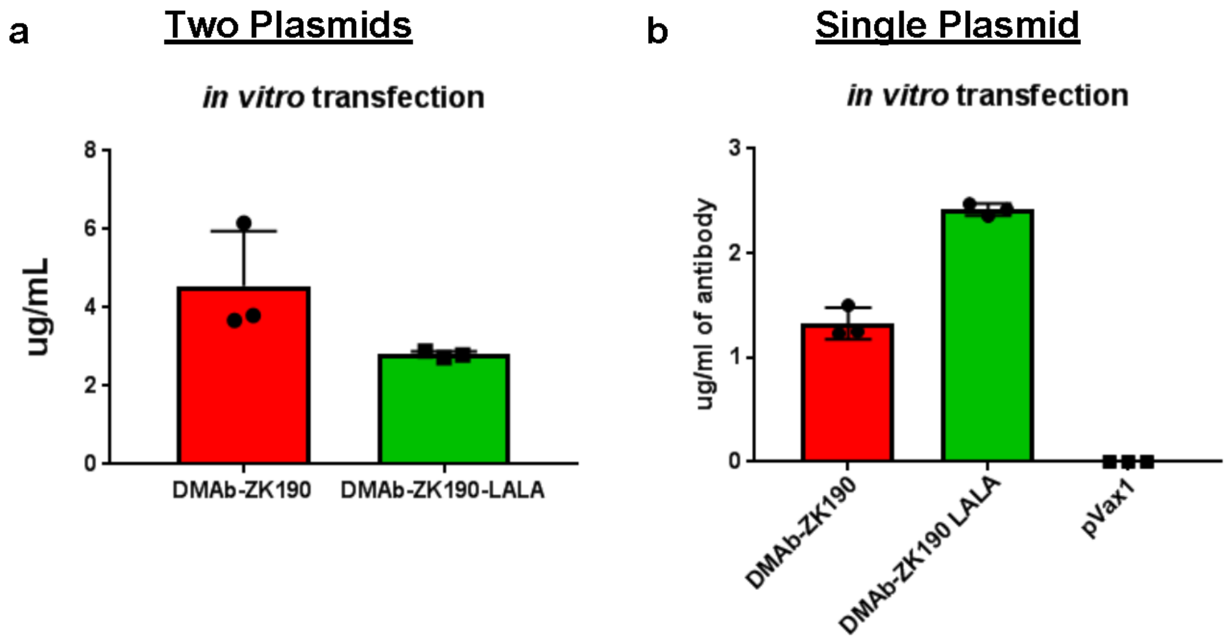

Supplemental Figure 1: **ZIKV DMAb expression in vitro.** In vitro expression of DMAb-ZK190 two plasmid (**a**) (n=3) or one plasmid (**b**) (n=3) system in transfected HEK 293 cells. Cell supernatant was harvested 48 hours post-transfection and human IgG1 expression was detected and quantified by ELISA. For each experiment "n" refers to biological replicates. Error bars refer to standard deviation.

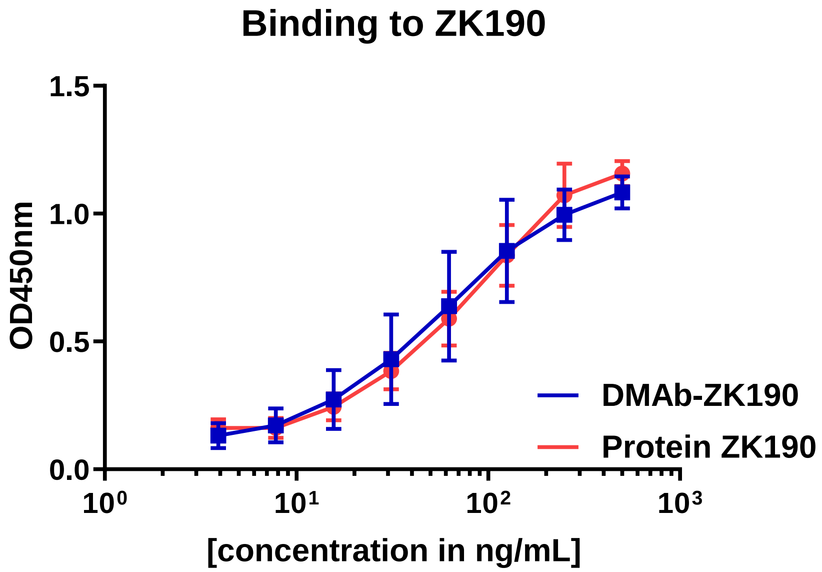

Supplemental Figure 2: **Quantification of ZIKV DMAb-ZK190 binding using recombinant ZK190 protein as a standard.** Sera from ZK190-DMAb injected animals (n=7) was quantified using recombinant ZK190 protein as a standard. Plates were coated with ZIKV E protein and detected as outline in the Binding ELISA methods section.

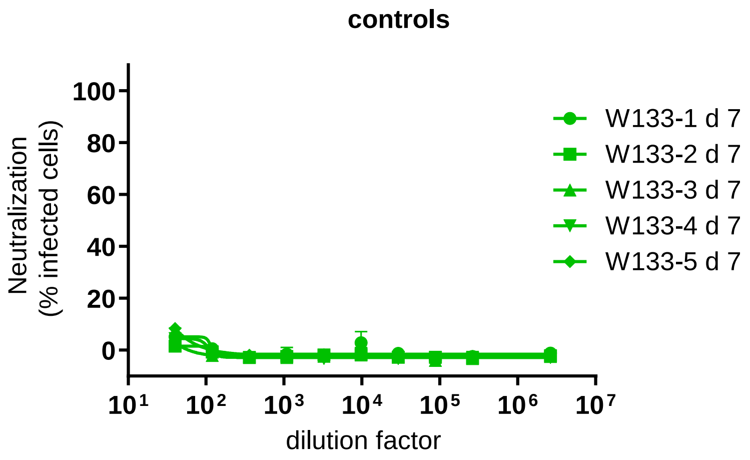

Supplemental Figure 3: *In vitro* neutralization activity of naïve mouse sera in a flow cytometry neutralization assay. Serial dilutions of sera from naïve mice (n=5) were evaluated in vitro in a flow-based assay for their ability to block ZIKV H/PF/2013 (100 p.f.u.) infection of Vero cells. Linear regression analysis was used to determine concentration of sera that neutralized infection by 50% compared to wells receiving virus only. For each experiment “n” refers to biological replicates.

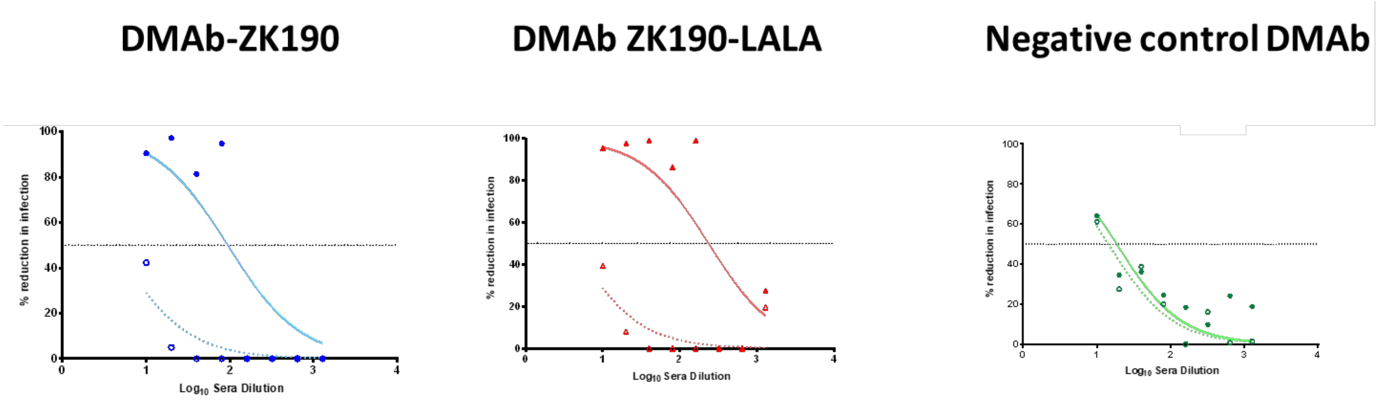

Supplemental Figure 4: *In vitro* neutralization activity of DMAb-ZK190 and DMAb-ZK190 LALA. (a) Serial dilutions of pooled day 7 sera from mice (n=5) injected with DMAb-ZK190, DMAb-ZK190 LALA, or a negative control DMAb were evaluated in an immunostaining-based assay for their ability to block ZIKV PR209 (100 p.f.u.) infection of Vero cells. Linear regression analysis used to determine concentration of DMAb in sera that neutralized infection by 50% (dotted line) compared to wells receiving virus only. For each experiment “n” refers to biological replicates.

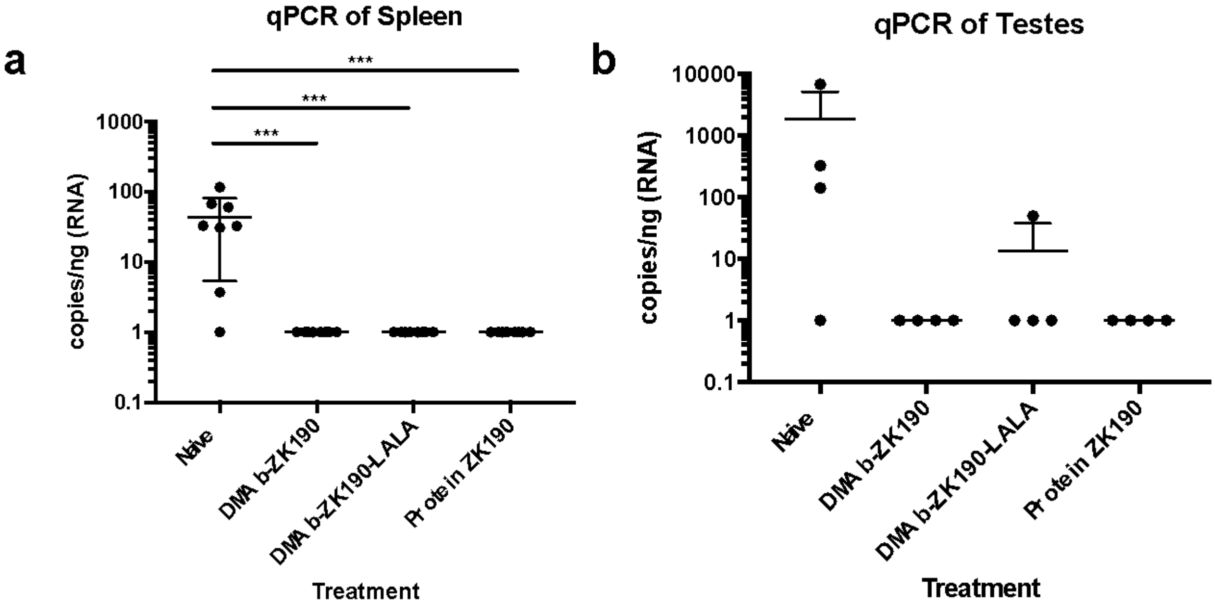

Supplemental Figure 5: Viral load in tissues following high dose ZIKV mouse challenge. Tissues were harvested from DMAb-ZK190, DMAb-ZK190-LALA, protein ZK190, and pVax11 control mice challenged with ZIKV (10^6 PFU dose). RNA was extracted from A) spleen (n=8) and B) testes (n=4) at the terminal endpoint. ZIKV genome copies/ng of RNA were detected by qRT-PCR. For each experiment "n" refers to biological replicates. Error bars refer to standard deviation.

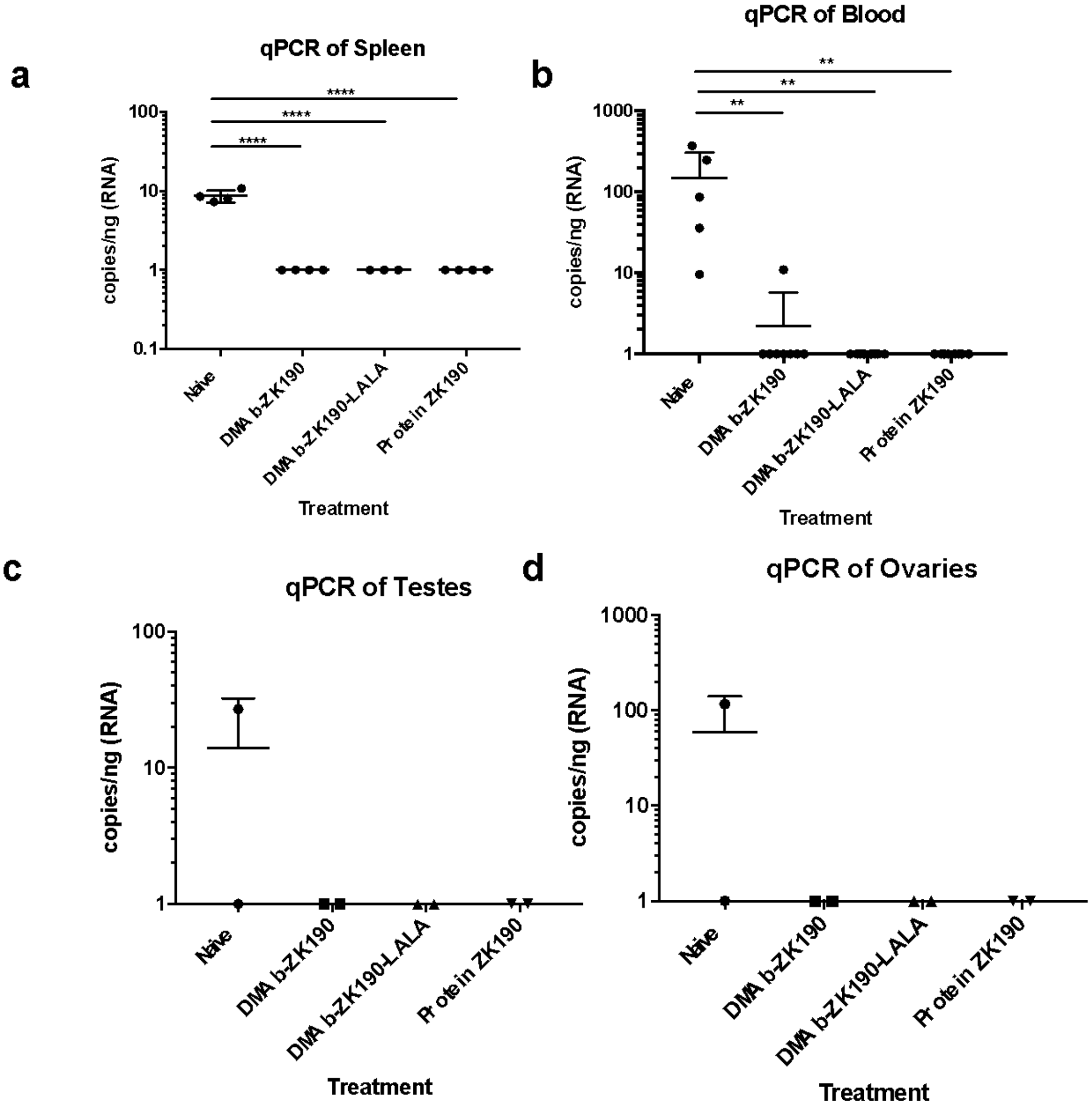

Supplemental Figure 6: Viral load in tissues following low dose ZIKV mouse challenge. Tissues were harvested from DMAb-ZK190, DMAb-ZK190-LALA, protein ZK190, and pVax11 control mice challenged with ZIKV (10^5 PFU dose). RNA was extracted from A) spleen (n=4, DMAb-ZK190, protein ZK190, pVax) (n=3 DMAb-ZK190-LALA), B) ovaries (n=2), C) testes (n=2) and D) blood (n=5, pVax)(n=8, DMAb-ZK190, DMAb-ZK190-LALA, protein ZK190) at

the terminal endpoint. ZIKV genome copies/ng of RNA were detected by qRT-PCR. For each experiment "n" refers to biological replicates. Error bars refer to standard deviation.

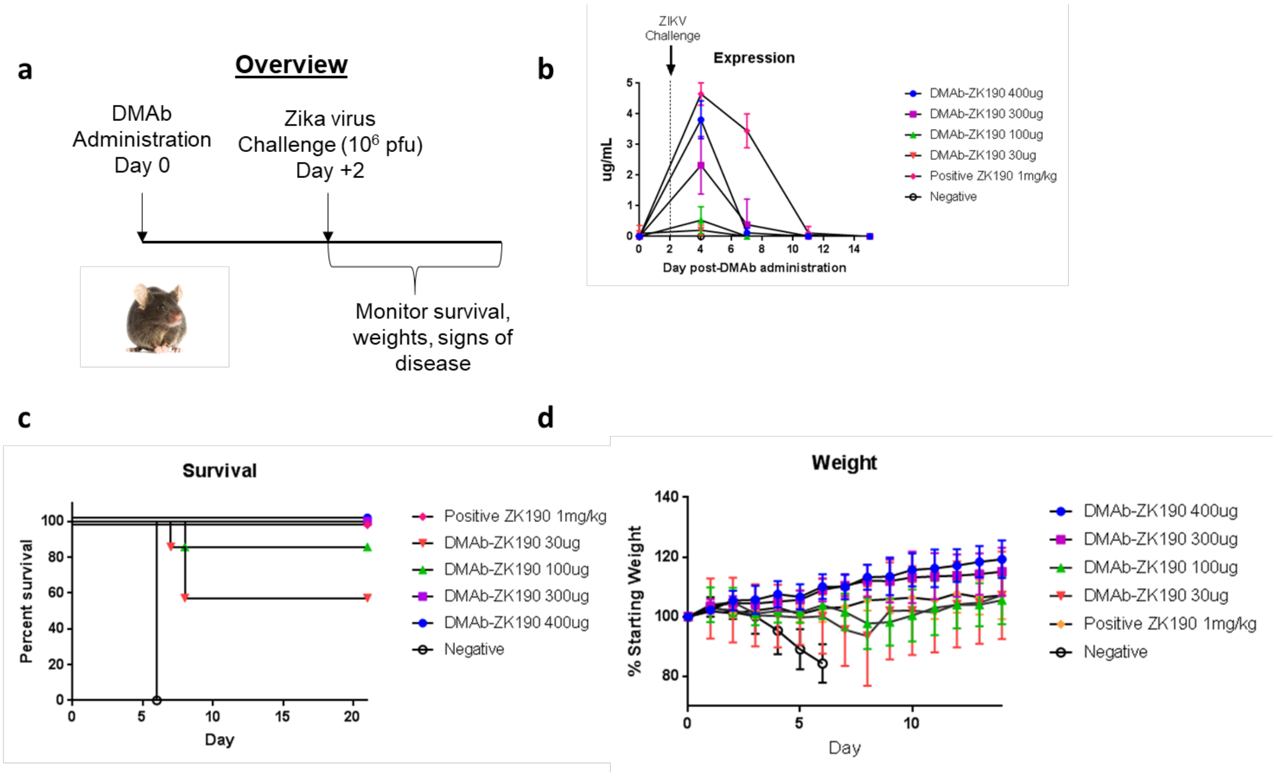

Supplemental Figure 7*: In vivo* protection by single plasmid DMAb-ZK190. **a**) Overview of the injection regimen. DMAbs were administered on day 0 and serum was collected on day 0, 4, 7, and 11 post-lethal challenge with 10^6 PFU of Zika Strain PR209. Animals were monitored for 21 days post-challenge for signs of disease and weight loss. **b**) Serum human IgG levels at day 0, 4, 7, and 11 post challenge. **c**) Survival of 30 ug, 100 ug, 300 ug, or 400 ug ZK190-DMAb receiving mice (n=7) compared to negative control (n=7) and protein IgG (n=7). **d**) Percentage weight change for negative control group receiving DMAb empty vector pVax11 (100 µg/mouse) compared to mice receiving treatment group ZK190 (30ug, 100ug, 300 µg or 400 ug) or protein ZK190 (1 mg/kg) For each experiment "n" refers to biological replicates. Error bars refer to standard deviation.

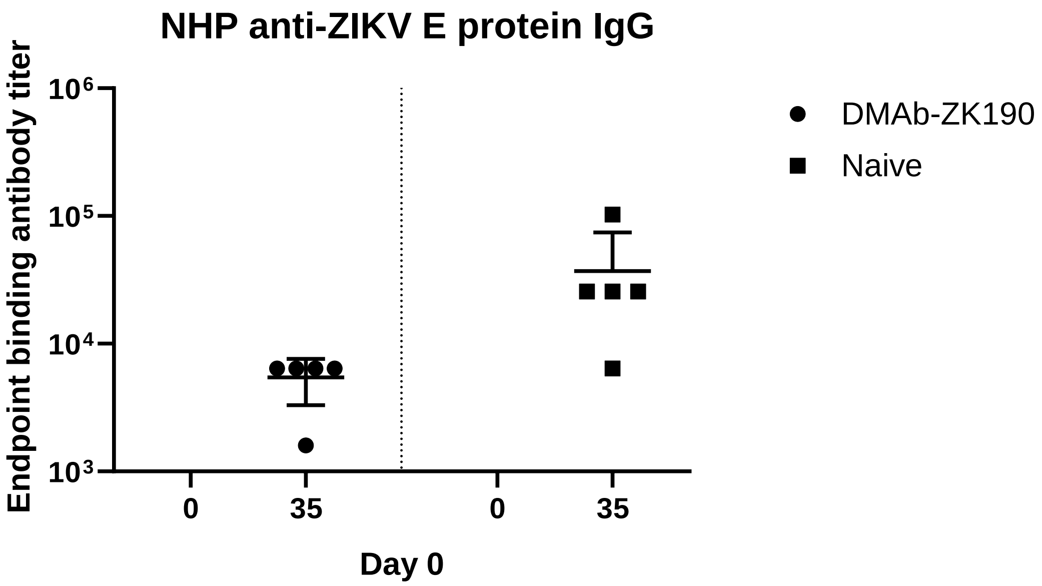

Supplemental Figure 8: Serum ZIKV endpoint titres in DMAb-ZK190 administered and naïve macaques following challenge. Endpoint binding antibody titres of naïve and ZK190-DMAb receiving NHPs at day 35 post-challenge as measured by binding ELISA on plates coated with ZIKV E protein (n=5).

Supplemental Table 1. Histopathology of testes of DMAb, protein or pVax treated mice post- Zika challenge.

Treatment Group	Histopathology
**DMAb-ZK190**	Testicle: Spermatogenesis<br>Epididymis: Maturing spermatids
**DMAb-ZK190-LALA**	Testicle: Spermatogenesis<br>Epididymis: Maturing spermatids
**Protein ZK190**	Testicle: Spermatogenesis<br>Epididymis: Maturing spermatids
**pVax 10^6 pfu**	Testicle: Spermatogenesis<br>Epididymis: Maturing spermatids
**pVax 10^5 pfu**	Testicle: Severe necrotizing and histiocytic orchitis with degeneration and regional parenchymal loss and edema (~40%)<br>Epididymis: Reduced sperm; multifocal luminal cell debris

Supplemental Table 2. Histopathology of ovaries of DMAb, protein or pVax treated mice post- Zika challenge.

Treatment Group	Histopathology
**DMAb-ZK190**	Uterus (myometrium): Mild granulocytic infiltrate<br>Ovary: multiple corpora lutea; folliculogenesis
**DMAb-ZK190-LALA**	Uterus (myometrium): Mild granulocytic infiltrate<br>Ovary: multiple corpora lutea; folliculogenesis
**Protein ZK190**	Uterus (myometrium): Mild granulocytic infiltrate<br>Ovary: multiple corpora lutea; folliculogenesis
**pVax 10^6 pfu**	Uterus (myometrium): Mild granulocytic infiltrate<br>Ovary: multiple corpora lutea; folliculogenesis
**pVax 10^5 pfu**	Mild glandular dilation with luminal debris; mild granulocytic infiltrate
