# Supplementary material for: In Vivo Delivery of a DNA-Encoded Monoclonal Antibody Protects Non-human Primates against Zika Virus
Source: Mol Ther. 2019 Apr 5;27(5):974–85. doi: 10.1016/j.ymthe.2019.03.005 (PMC6520333; doi:10.1016/j.ymthe.2019.03.005)
Supplement: Document S2. Article plus Supplemental Information [file mmc2.pdf]

Molecular Therapy
Original Article

In Vivo Delivery of a DNA-Encoded Monoclonal Antibody Protects Non-human Primates against Zika Virus

Rianne N. Esquivel,[1,5] Ami Patel,[1,5] Sagar B. Kudchodkar,[1] Daniel H. Park,[1] Karin Stettler,[2] Martina Beltramello,[2] Jeffrey W. Allen,[3] Janess Mendoza,[3] Stephanie Ramos,[3] Hyeree Choi,[1] Piyush Borole,[1] Kanika Asija,[1] Mamadou Bah,[1] Shareef Shaheen,[1] Jing Chen,[3] Jian Yan,[3] Amy C. Durham,[4] Trevor R.F. Smith,[3] Kate Broderick,[3] Ghiabe Guibinga,[3] Kar Muthumani,[1] Davide Corti,[2] Laurent Humeau,[3] and David B. Weiner[1]

[1]Vaccine & Immunotherapy Center, The Wistar Institute of Anatomy & Biology, Philadelphia, PA, USA; [2]Humabs BioMed: a subsidiary of Vir Biotechnology, Bellinzona, Switzerland; [3]Inovio Pharmaceuticals, Plymouth Meeting, PA, USA; [4]Department of Pathobiology, School of Veterinary Medicine, University of Pennsylvania, Philadelphia, PA, USA

Zika virus (ZIKV) infection is endemic to several world regions, and many others are at high risk for seasonal outbreaks. Synthetic DNA-encoded monoclonal antibody (DMAb) is an approach that enables *in vivo* delivery of highly potent mAbs to control infections. We engineered DMAb-ZK190, encoding the mAb ZK190 neutralizing antibody, which targets the ZIKV E protein DIII domain. *In vivo*-delivered DMAb-ZK190 achieved expression levels persisting >10 weeks in mice and >3 weeks in non-human primate (NHPs), which is protective against ZIKV infectious challenge. This study is the first demonstration of infectious disease control in NHPs following *in vivo* delivery of a nucleic acid-encoded antibody, supporting the importance of this new platform.

INTRODUCTION

Zika virus (ZIKV) is a mosquito-borne infection that has become an important global public health concern, with over 2 billion people at risk. ZIKV infection carries significant risks during pregnancy resulting in severe developmental defects in newborns, including microcephaly and severe cognitive impairment. Guillain-Barré syndrome and other neurological symptoms have also been observed in a subset of infected individuals.[1] Immune-privileged sites such as the testes[2,3] and brain[4,5] can harbor ZIKV. Harboring in the testes can lead to potential transmission through sexual contact months after convalescence.[6] Furthermore, ZIKV infection can drive severe pathology in the testes in animal models.[7,8] Consequently, rapid preventative interventions for ZIKV are a pressing global need for people living in endemic countries, travelers, and other high-risk populations.

Individuals who recover from infection develop ZIKV-specific, protective antibodies, and passive transfer of sera from naturally infected or vaccinated individuals protects mice against lethal ZIKV infection.[9] Consequently, several monoclonal antibodies (mAbs) with potent neutralizing activity have been isolated from convalescent donors, with further demonstration of protection against ZIKV infection in mouse and non-human primate (NHP) models.[10–13] Recombinant mAbs are, therefore, a highly promising tool for study of the prevention of this important emerging infectious disease. While important, the uptake of mAb biologics for prophylaxis in large global populations spread across developed and developing countries alike is challenging due to delivery and manufacturing limitations and a requirement for cold-chain storage. *In vivo* delivery of synthetic nucleic acid expression vectors encoding engineered mAb genes represents a possible alternative novel approach, with great potential to alleviate the critical challenges with recombinant mAb biologics.

We engineered synthetic plasmid DNA-encoding mAb (DMAb) cassettes expressing the potent anti-ZIKV mAb ZK190 (DMAb-ZK190), a clone that binds uniquely to the ZIKV E antigen and is protective in mice,[11] and also we engineered a variant, DMAb-ZK190-LALA, designed to abrogate Fc receptor (FcR) binding. DMAbs were administered *in vivo* to mice and rhesus macaques through intramuscular (IM) administration facilitated by adaptive constant current electroporation (CELLECTRA), resulting in *in vivo* immunoglobulin (Ig) production and secretion of functional mAb in circulation for several months. When animals were challenged, these engineered DMAbs provided rapid protection against ZIKV infection and pathogenesis first in mice, protecting from infection and preventing damage in the immune-privileged testes. Engineered DMAb-ZK190 was also expressed in NHPs and protected rhesus macaques against ZIKV strain PRVABC59 infection, displaying a dramatic protection against viral load. To our knowledge, this is the first demonstration of *in vivo* expression and prevention of infection with a nucleic acid-encoded antibody in a NHP model. Taken together, the data support further

Received 11 October 2018; accepted 11 March 2019;
https://doi.org/10.1016/j.ymthe.2019.03.005.

[5]These authors contributed equally to this work.

Correspondence: David B. Weiner, Vaccine & Immunotherapy Center, The Wistar Institute of Anatomy & Biology, 3601 Spruce Street, Philadelphia, PA 19104, USA.
E-mail: dweiner@wistar.org

 © 2019 The Author(s).
This is an open access article under the CC BY license (http://creativecommons.org/licenses/by/4.0/).

study of DMAb delivery for the prevention of ZIKV and other infectious diseases.

RESULTS

Engineering of DMAb-ZK190 and DMAb-ZK190-LALA DNA-Encoded mAbs

mAb clone ZK190 was isolated from human peripheral blood mononuclear cells (PBMCs) following ZIKV infection.[11] ZK190 binds to the ZIKV E protein DIII domain, and it binds in a unique conformation on the 5-fold vertex compared to other identified mAbs,[11] enabling full occupancy of all 180 E proteins. The mAb pulls the viral envelope away from the virion surface and disrupts the particle.[14] ZK190 and variant ZK190-LALA, designed with L234A and L235A mutations to prevent Fc gamma receptor interactions, both demonstrated strong protection in mice.[14]

The ZK190 heavy chains (HCs) and light chains (LCs) were engineered into both a dual-plasmid and single-plasmid DNA DMAb platform. Delivery of the single plasmid or co-delivery of the two plasmids results in expression of full-length human IgG1 DMAb-ZK190 or DMAb-ZK190-LALA. DMAb plasmid expression was first tested *in vitro*. A quantitative ELISA was performed on cell supernatants following transfection of HEK293T cells with DMAb-ZK190 or DMAb-ZK190-LALA to confirm plasmid expression and IgG secretion (Figure S1).

DMAb-ZK190 and DMAb-ZK190-LALA Express *In Vivo* and Bind to Target ZIKV E Protein

C57BL/6 mice were injected with dual-plasmid construct DMAb-ZK190 (200 μg) or dual-plasmid construct DMAb-ZK190-LALA (200 μg) and hyaluronidase by IM injection, followed by electroporation (IM-EP, CELLECTRA) delivery. Peak expression levels for DMAb-ZK190 and DMAb-ZK190-LALA in mouse sera, as measured by ELISA detecting human IgG, reached a mean of 27.0 μg/mL (±2.6 SEM) and 62.1 μg/mL (±6.4 SD), respectively. Notably, significant human IgG1 expression persisted 10 weeks (Figures 1A and 1B) and longer, indicative of the *in vivo* stability of mAb expression from a DNA plasmid.

Sera collected on day 7 from DMAb-ZK190- and DMAb-ZK190-LALA-administered mice bound to recombinant ZIKV E protein. Binding was first assessed by ELISA on dilutions of DMAb-administered mouse serum (n = 5 mice/group), in comparison with recombinant protein ZK190 (Figures 1C and 1D). Half-maximum effective concentrations (EC_{50}) were calculated, demonstrating DMAb-ZK190 and DMAb-ZK190-LALA equivalency in binding capacity to their recombinant mAb counterpart. We compared DMAb-ZK190 binding with recombinant ZK190 as a standard, demonstrating identical binding to ZIKV E protein (Figure S2).

Binding activity was further confirmed by western blots loaded with ZIKV E protein and probed with equal concentrations of serum (13 ng/mL) from DMAb-administered mice (Figures 1E and 1F).

DMAb-ZK190 and DMAb-ZK190-LALA Neutralize ZIKV Strains H/PF/2013 and PR209

Aliquots of the same day 7 sera from DMAb-ZK190- and DMAb-ZK190-LALA-administered mice were evaluated for their ability to neutralize ZIKV infection in *in vitro* microneutralization assays. A flow cytometry-based microneutralization assay was performed utilizing dilutions of sera from DMAb-administered mice and ZIKV strain H/PF/2013 (MOI of 0.35). After 4 days, the cells were fixed and stained with the anti-flavivirus mouse mAb 4G2. The cells were incubated with a goat anti-mouse IgG conjugated to Alexa Fluor488 (Jackson ImmunoResearch Laboratories, 115485164) and analyzed by flow cytometry. We observed similar results, with DMAb-ZK190 and DMAb-ZK190-LALA exhibiting comparable neutralization half-maximum inhibitory concentration (IC_{50}) titers to the corresponding ZK190 IgG control (Figures 1G and 1H; Figure S3).

In parallel, a series of 2-fold serial dilutions of pooled DMAb-ZK190, DMAb-ZK190-LALA, or pVax1 sera collected at 7 days post-infection was pre-incubated with ZIKV (strain PR209) for 1.5 h and then added to confluent Vero cells. 4 days later, cells were fixed and infected cells were identified by immunostaining with a pan-flavivirus mAb. The percent infected cells for each serum dilution was compared to the percent infected cells in wells that received virus alone.

Variable slope, non-linear regression analysis was used to identify the dilution of each serum expected to neutralize infection by 50% (microneutralization 50 [MN_{50}]). The DMAb-ZK190 day 7 serum neutralized PR209 infection with a MN_{50} dilution factor of 97, which corresponded to a serum DMAb IgG level of 10.5 ng/mL (Figure S4). The DMAb-ZK190-LALA day 7 serum also neutralized infection, with an MN_{50} dilution factor of 196, corresponding to a serum DMAb IgG concentration of 16.2 ng/mL. No appreciable neutralization activity was seen in pooled day 0 sera from either group or in serum from pVax1-injected mice. Taken together, these results indicate that the *in vivo* DMAb-produced ZK190 is potent *in vivo*, maintaining its functionality for neutralizing ZIKV infection.

DMAbs Protect Mice against Lethal High-Dose ZIKV Challenge in IFNAR$^{-/-}$ Mice

To assess *in vivo* functionality, DMAb-ZK190 and DMAb-ZK190-LALA protective efficacy was evaluated in the IFNAR$^{-/-}$ lethal ZIKV mouse challenge model as previously described.[15] IFNAR$^{-/-}$ mice were administered DMAb-ZK190 (200 μg), DMAb-ZK190-LALA (200 μg) plasmid DNA, or negative control vector pVax1 (200 μg) via IM-EP injection. At 2 days post-DMAb administration, mice were challenged with a lethal dose of ZIKV virus (strain PR-209, 10^6 plaque-forming unit [PFU]/mouse) (Figure 2A). For direct *in vivo* comparison with recombinant protein, mAb ZK190 IgG (1 mg/kg) was delivered intraperitoneally (i.p.) to a parallel group of mice 1 day prior to infection. DMAb expression levels assayed at day +2 following ZIKV infection were 7.9–26.7 μg/mL for DMAb-ZK190 and 8.5–39.1 μg/mL for DMAb-ZK190-LALA (Figure 2B). Both

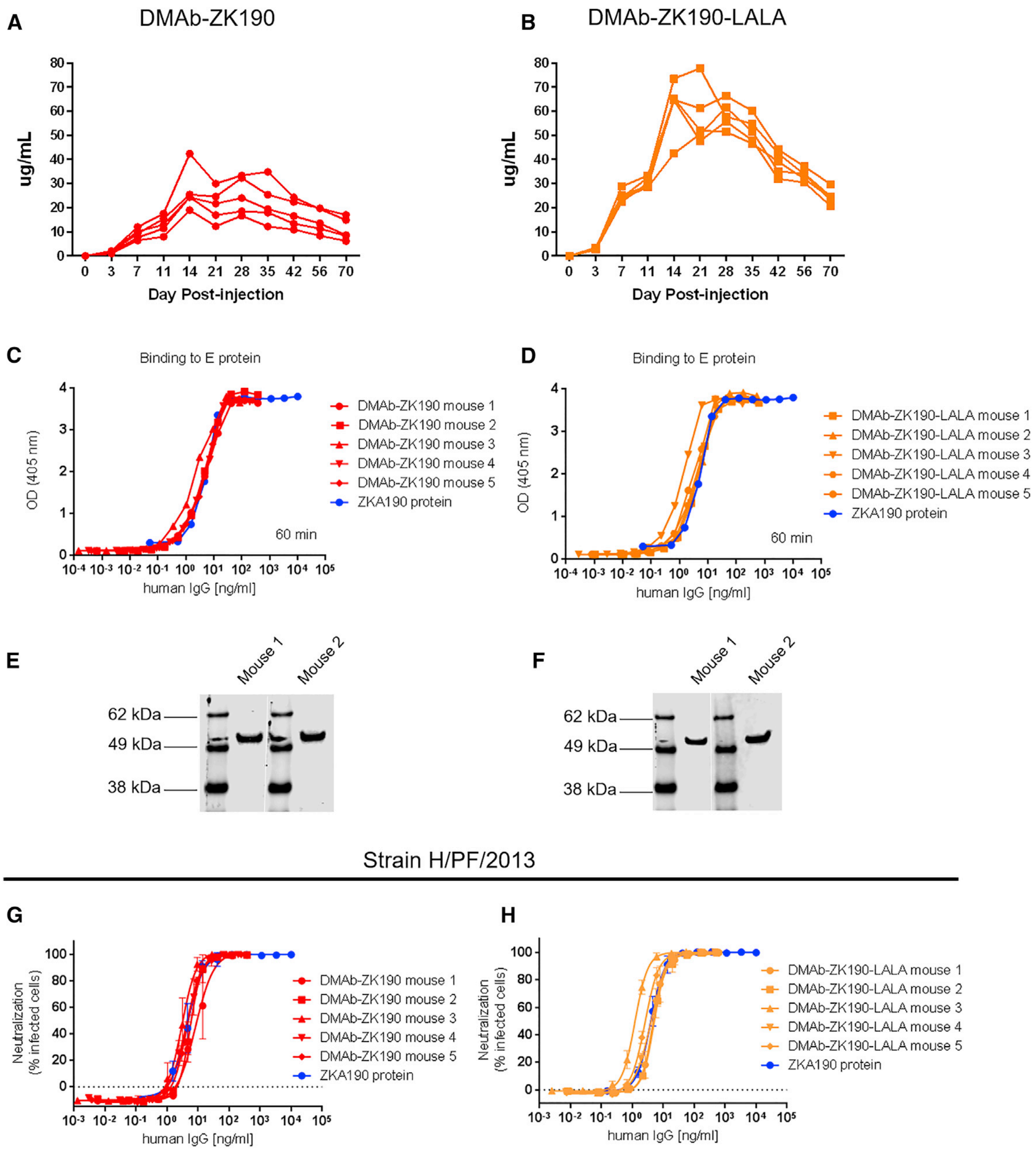

Figure 1. *In Vivo* DMAb-ZK190 and DMAb-ZK190-LALA Pharmacokinetic Expression, Binding to ZIKV E Protein, and Neutralization Activity

Conditioned C57BL/6 mice were injected with a 200 μg dual-plasmid construct of either ZK190 (A) or ZK190-LALA (B) (n = 5). Human IgG1 was monitored in mouse serum for >70 days. Serum samples from mice administered DMAb-ZK190 and DMAb-ZK190-LALA were evaluated to confirm binding to ZIKV E protein (C–F). DMAb expression is compared with protein IgG by binding to ZIKV E protein by ELISA (C and D) and western blot loaded with Zika E protein. Western blots were cropped for clarity (white bar) (E and F) and probed with serum from DMAb-administered mice. For each experiment "n" refers to biological replicates. (G and H) Serial dilutions of day 7 sera from (G) DMAb-ZK190-injected and (H) DMAb-ZK190 LALA mice were evaluated *in vitro* in a flow-based assay for their ability to block ZIKV H/PF/2013 (100 pfu) infection of Vero cells. Protein ZK190 mAb or ZK190 LALA mAb were included as controls. Linear regression analysis was used to determine concentration of DMAb in sera that neutralized infection by 50% compared to wells received virus only. For each experiment, "n" refers to biological replicates.

DMAb-ZK190 and DMAb-ZK190-LALA provided 100% protection against mortality and signs of morbidity, comparable to the positive IgG control group (Figures 2C–2E). All negative control animals receiving pVax1 plasmid succumbed to disease. Consistent with the lack of morbidity and weight loss in mice receiving DMAb or recombinant mAb following ZIKV challenge, a significantly lower viral load

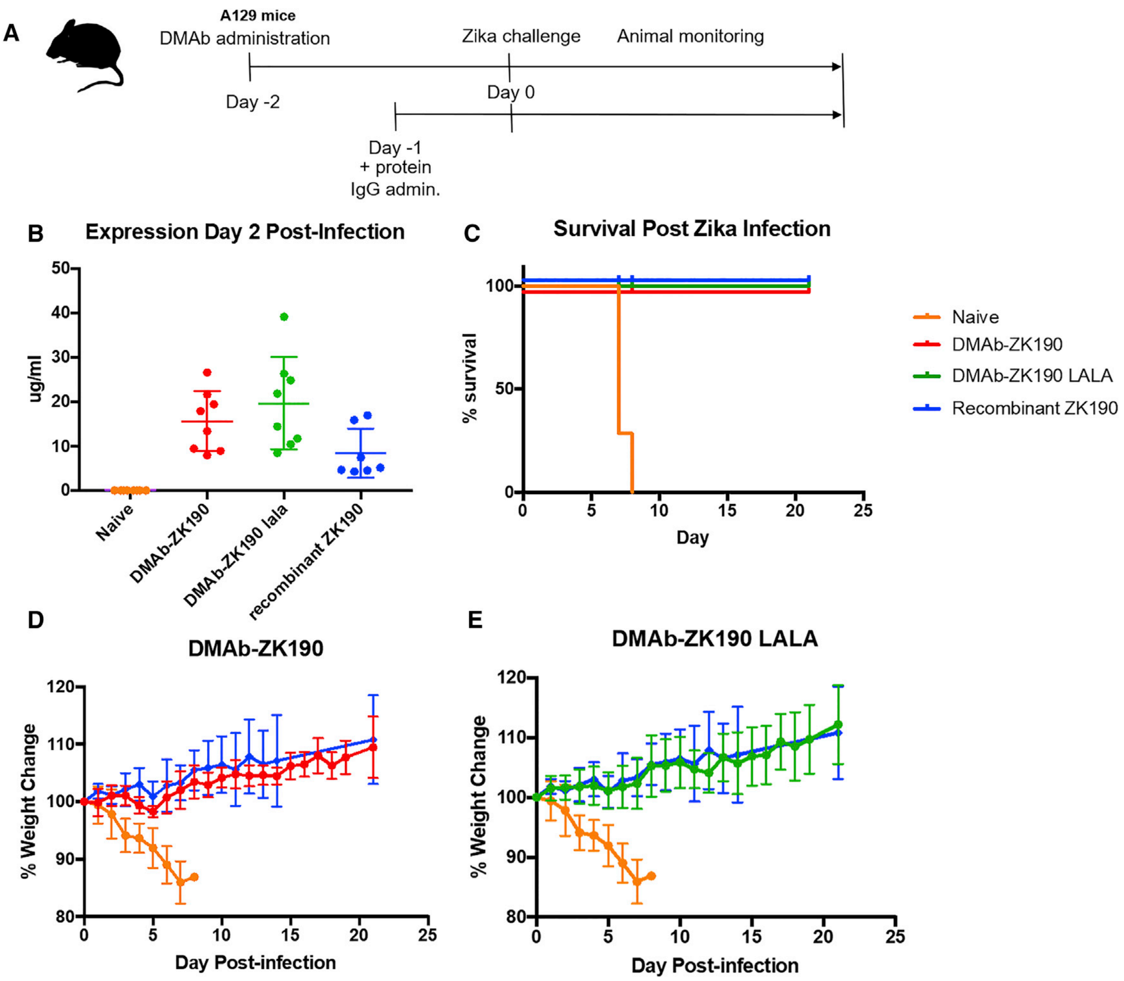

Figure 2. *In Vivo* Protection by DMAb-ZK190 and DMAb-ZK190-LALA

(A) Overview of the injection regimen. DMAbs were administered on day −2, and serum was collected on day 2 after lethal challenge with 10^6 PFU Zika Strain PR209. Animals were monitored for 21 days post-challenge for signs of disease and weight loss. (B) Serum human IgG levels at day 2 post-challenge. (C) Survival of ZK190 and ZK190-LALA DMAb-receiving mice (n = 8) compared to negative control (n = 8) and protein IgG (n = 6). (D and E) Percentage weight change for negative control group receiving DMAb empty vector pVax1 (100 μg/mouse) compared to mice receiving treatment group ZK190-LALA (300 μg) (D), ZK190 (300 μg) (E), or protein ZK190 (1 mg/kg). For each experiment, "n" refers to biological replicates. Error bars refer to SD.

was noted in the spleen (Figure S5) compared with naive virally challenged animals. Additionally, while a range of viral loads, reaching 10,000 copies/ng RNA, was detected in the testes of naive infected mice, of the DMAb- and protein IgG-receiving mice, only one DMAb-ZK190-LALA mouse had a detectable viral load (Figure S5).

Zika DMAbs Protect Mouse Testes from Damage in a Low-Dose Challenge in IFNAR$^{-/-}$ Mice

In humans, ZIKV antigen can be detected in immune-privileged sites, long after infection has been cleared from peripheral circulation.[16] In mice, a low-dose, sublethal challenge allows survival of negative control mice, allowing study of the long-term impact of ZIKV on organ pathology.[8] We repeated the ZIKV challenge study described above with a sublethal 10^5-PFU dose of ZIKV (strain PR209). IFNAR$^{-/-}$ mice (n = 8/group) were administered DMAb-ZK190 (200 μg), DMAb-ZK190-LALA (200 μg), or control pVax1 by IM injection followed by IM-EP. 2 days later, mice were challenged with ZIKV (10^5 PFU). Recombinant ZK190 mAb was administered i.p. to a separate group (n = 8) 1 day prior to infection (Figure 3A). Expression levels of DMAb-ZK190 and DMAb-ZK190-LALA were 4–12 and 4.6–11.1 μg/mL, respectively, on day 2 following ZIKV challenge (Figure 3B). DMAb-ZK190, DMAb-ZK190-LALA, and protein ZK190 groups were completely protected from weight loss and signs of diseases, whereas the negative control group experienced significant weight loss (Figures 3D and 3E). A significantly lower viral load was detected in the spleen and blood (Figure S6) of DMAb or recombinant mAb-treated mice compared with naive animals.

Importantly, IFNAR$^{-/-}$ mice administered DMAb-ZK190, DMAb-ZK190-LALA, or recombinant ZK190 displayed no lesions within

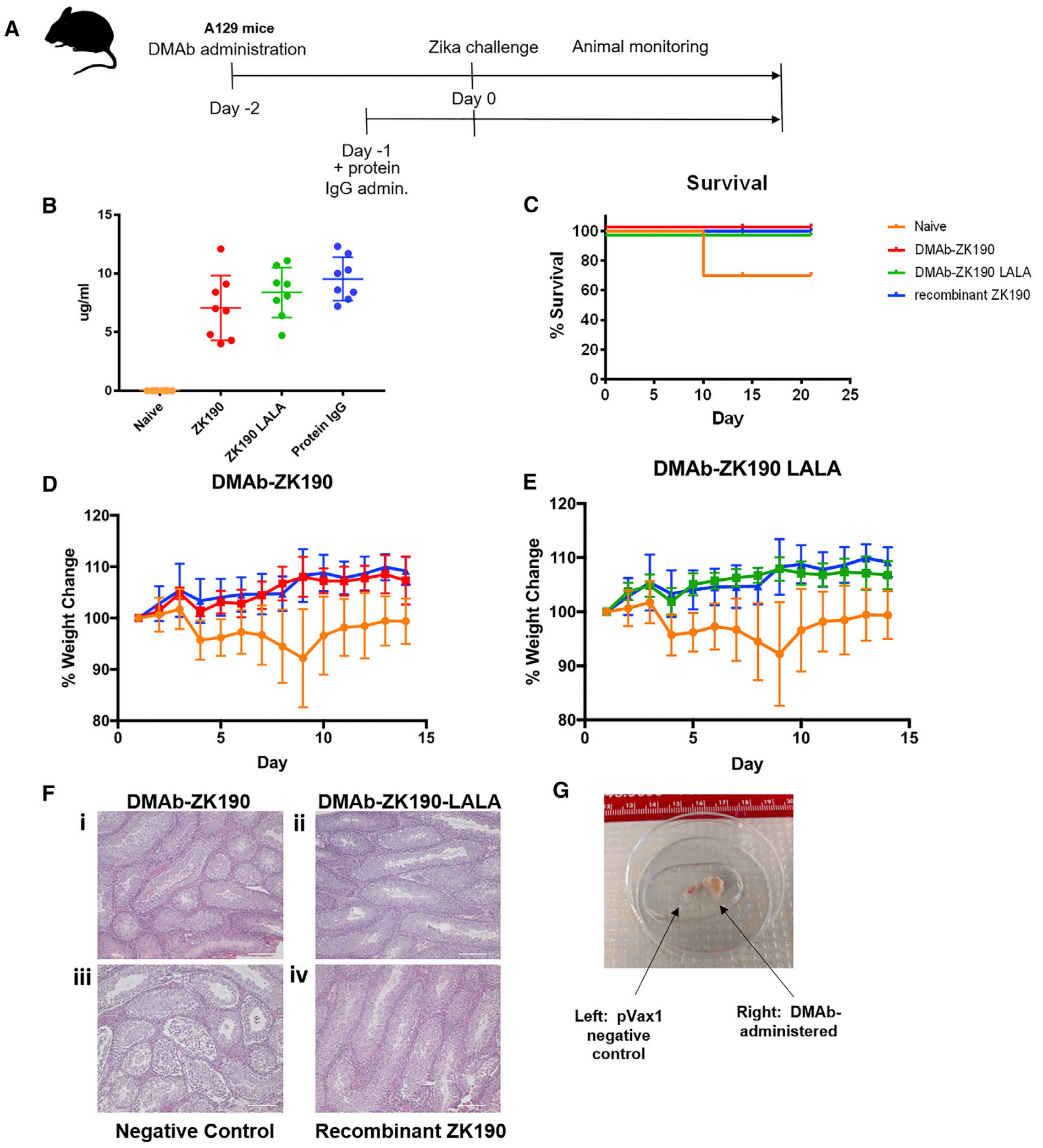

Figure 3. *In Vivo* Protection of Mouse Testes by DMAb-ZK190 and DMAb-ZK190-LALA in Low-Dose Challenge

(A) Overview of the injection regimen. DMAbs were administered on day −2, and serum was collected on day 2 after lethal challenge with 10^5 PFU Zika Strain PR209. Animals were monitored for 21 days post-challenge for signs of disease and weight loss. (B) Serum human IgG levels at day 2 post-challenge. (C) Survival of ZK190 and ZK190-LALA DMAb-receiving mice (n = 8) compared to negative control (n = 8) and protein IgG (N = 6). (D and E) Percentage weight change for negative control group receiving DMAb empty vector pVax1 (100 μg/mouse) compared to mice receiving treatment group ZK190-LALA (300 μg) (D), ZK190 (300 μg) (E), or protein ZK190 (1 mg/kg). (F) Testes sections from pVax1- and DMAb-treated groups were collected 21 days after challenge and stained with H&E for histology. The sections taken from representative, unprotected pVax1 control animals show pathology. Scale bar, 100 μm. (G) Whole testes from pVax1- (left) or ZK190 DMAb- (right) treated mice. Ruler displays centimeters. For each experiment "n" refers to biological replicates. Error bars refer to SD.

the testes histologically, whereas the negative control mice developed severe testicular lesions (Figure 3F, i–iv; Table S1). The negative control animals that survived the low 10^5-PFU dose challenge grossly exhibited significant testicular atrophy (Figure 3G). Histological sections were evaluated by a blinded board-certified veterinary pathologist: reported findings for the negative control animals included severe interstitial edema, parenchymal loss, necrosis of the testes and reduction in sperm, and necrosis in the epididymis (Figure 3F).

The non-DMAb-injected challenged control testes were severely damaged, extending previous data reported in the literature.[8,17] ZIKV was also detected in the testis or ovary of the naive control challenge mice of one naive infected mouse, but not in the DMAb and protein Ig groups (Figure S6). By comparison, DMAb-ZK190- and DMAb-ZK190-LALA-treated animals were completely protected, showing no signs of infection in the testes or epididymis (Table S1). Ovaries from DMAb-treated and negative animals were evaluated, but no signs of ZIKV damage were observed (Table S2).

Zika DMAbs Protect Rhesus Macaques against ZIKV Challenge (PRVABC59)

Based on the promising protection in mouse models, we evaluated DMAb-ZK190 expression and protection in a rhesus macaque ZIKV challenge model. In other studies, there is a trend toward better protective efficacy with recombinant ZK190 in mice, compared with recombinant ZK190-LALA.[14] Based on these studies and the promising protection data with DMAb-ZK190 in mice, we selected this construct to move forward into NHP study. Rhesus macaques (n = 5) received 3 sequential 6-mg injections (18 mg total DNA) of DMAb-ZK190 by IM-EP administration on days −10, −7, and −4. For the simplicity of delivery and to closely model translational use in humans, a single-plasmid system was used. A mouse study with different doses of the single-plasmid construct was performed to confirm *in vivo* expression (Figure S7) and protection against lethal high-dose ZIKV challenge. Macaques were challenged 10 days after the first DMAb injection with 10^4 PFU ZIKV (strain PRVABC59), in parallel with a naive control group (n = 5) (Figure 4A). We detected DMAb-ZK190 expression ranging from 200 to 800 ng/mL on the day of challenge (Figure 4B). ZIKV infection is not lethal in NHPs, therefore, we monitored viral load over 28 days in all challenged animals. We observed a reduction in viral loads in 4/5 animals in the DMAb-ZK190 group and a marked delay in infection in the last animal (Figure 4D). As expected, no DMAb expression was present in the control animals (Figure 4B), and viral load was detected in all 5 animals (Figure 4E).

We assayed anti-ZIKV E protein endpoint binding titers for all challenged animals from sera obtained on day 35 post-challenge. We observed lower anti-E protein total IgG antibody titers in the DMAb-administered animals (Figure S8), further supporting the lower viremia in DMAb-ZK190 animals.

DISCUSSION

Our studies describe the first protection with an *in vivo*-delivered anti-ZIKV DMAb (DMAb-ZK190) in a rhesus macaque model of ZIKV infection. To our knowledge, this is the first successful demonstration of protection against ZIKV, or any infectious disease, with a nucleic acid-encoded antibody in a NHP model. ZIKV infection has become endemic in many regions of the world, and strategies for direct *in vivo* delivery of highly potent mAbs, like DMAbs, would be valuable for conferring rapid, transient preventative protection against ZIKV infection in high-risk populations. DMAb-ZK190 protection in the rhesus macaque model is an important step forward for this technology and for further translation of this approach to the clinic.

Sexual transmission of ZIKV is well documented, and approaches to prevent infection in immune-privileged sites are critical to halting human-to-human transmission and vertical transmission from mother to child. In our studies, we demonstrated protection in mice against the highly pathogenic ZIKV strain, PR-209. DMAb-ZK190- and DMAb-ZK190-LALA-administered animals were completely protected against testicular damage and atrophy after either high- or low-dose challenges, with histologically normal testes, characterized by normal spermatogenesis and maturing spermatids. Untreated control animals in the low sublethal dose group displayed severe destruction of the testes and epididymis, including severe organ atrophy with parenchymal loss, edema, necrosis, and loss of maturing sperm. Importantly, analysis of viral loads in multiple tissues demonstrates that DMAb delivery protected against systemic and disseminated infection to multiple organs. DMAb delivery expresses for several months, significantly extending the duration of recombinant mAb in serum. Our study further extends the data on ZK190, demonstrating protection in male mice and, importantly, protection against severe infection in rhesus macaques.

ZIKV epidemiology overlaps with other flaviviruses, including Dengue virus (DENV), yellow fever virus, and West Nile virus.[18–21] While antibody enhancement antibody-dependent enhancement (ADE) is an important concern for Dengue virus immunization and infection, the relevance of ADE for ZIKV is unclear. The diversity of ZIKV strains is <1%,[22] and there is no evidence in NHPs or humans for ZIKV versus ZIKV ADE or even ZIKV versus Dengue virus ADE.[23,24] Observed ADE *in vitro* and in mice may be related to experimental design.[25] While more study in this area is likely necessary, we also evaluated DMAb-ZK190-LALA to mitigate any conceptual or potential risk that may be associated with such a DMAb delivery as well as proof of principle for such ADE phenomena. In this regard, we observed no difference in protective efficacy between DMAb-ZK190 and DMAb-ZK190-LALA in mice.

We observed that protection by DMAb-ZK190 in NHPs was not associated with any enhancement of infection. Even at low serum levels, DMAb-ZK190 had a positive effect on controlling infection in 5/5 animals and significantly lowered viral loads in 4/5 NHPs. We further show that DMAb-ZK190-administered animals have lower anti-ZIKV E protein total IgG titers, further supporting DMAb efficacy. DMAbs delayed the course of infection, and the detected viral load is likely the result of a host anti-DMAb antibody response and not due to lack of efficacy; however, further study of possible ZIKV escape mutants would be highly informative. Additional DMAb cocktail development targeting multiple ZIKV E protein domains may provide more sterilizing protection. NHPs are known to develop anti-human ADA against human mAbs,[26] and it is anticipated that a human DMAb would have minimal immunogenicity in the human host.

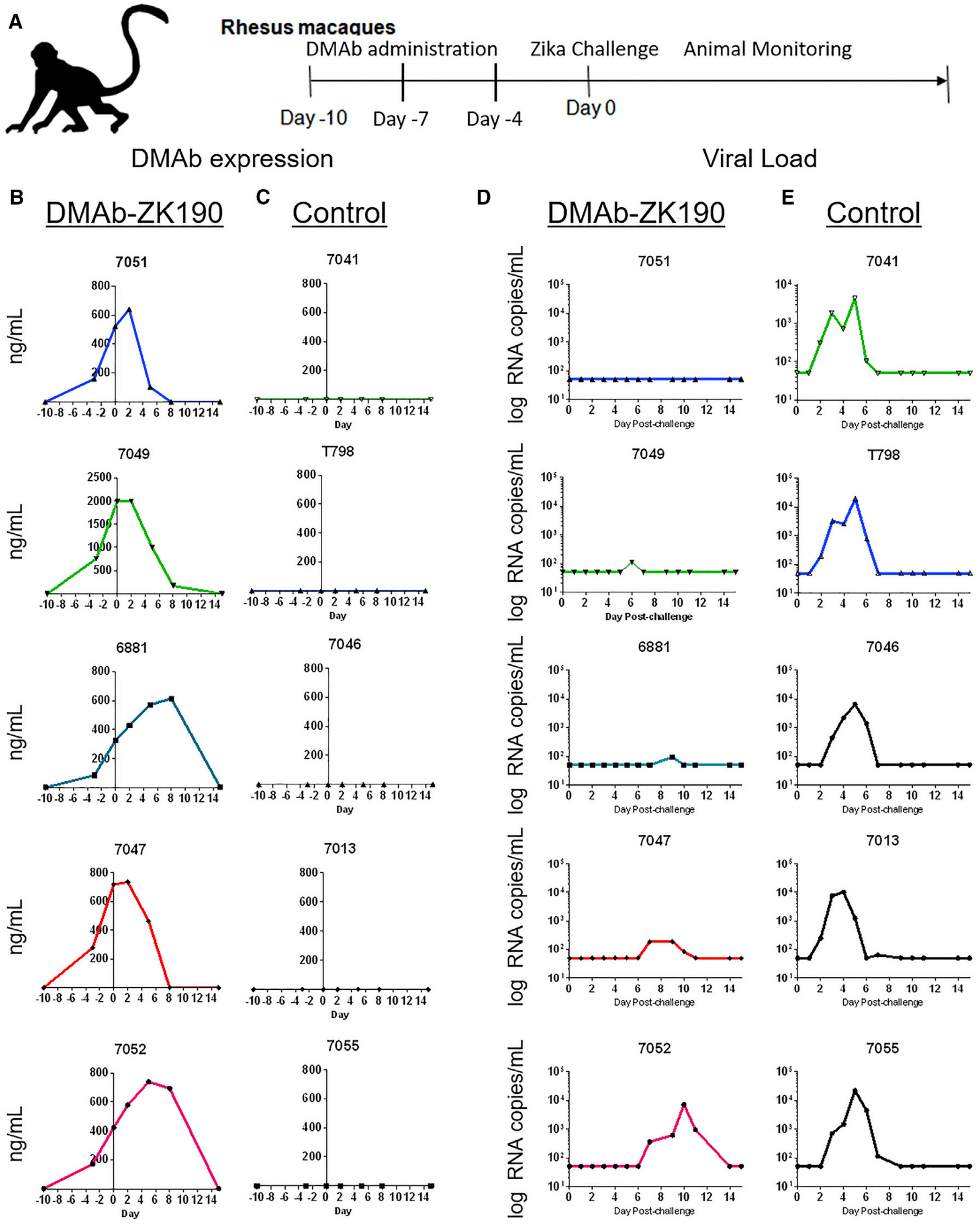

Figure 4. *In Vivo* Protection against ZIKV Challenge in Rhesus Macaques following the Administration of DMAb-ZK190 or Naive Control

(A) Overview of the injection regimen in rhesus macaques. DMAbs were administered in 3 sequential administrations on days 0, 3, and 6, and serum was collected serially throughout the study. Macaques were challenged with 10^4 PFU ZIKV strain PRVABC59 on day 0. (B) DMAb-ZK190 (n = 5) serum human IgG levels during the course of the challenge experiment. (C) Naive control (n = 5) serum human IgG levels during the course of the challenge experiment. (D) Serum ZIKV viral loads in DMAb-ZK190-administered macaques following challenge (n = 5). (E) Serum ZIKV viral loads in naive control macaques following challenge (n = 5). For each experiment, "n" refers to biological replicates.

We evaluated the expression of both single-plasmid and dual-plasmid constructs in mice, demonstrating protective efficacy even at low doses of the DMAb-ZK190 single-plasmid construct (Figure S7). Importantly, a single construct would be simpler for clinical translation, therefore, we moved the single plasmid into NHP study. It is likely that, similar to mice, higher expression levels would be observed with two-plasmid delivery in NHPs. Further studies investigating plasmid structure among other strategies to further increase expression levels in larger animals are important. A larger study would also provide more clarity on antibody concentration trough levels in NHPs. Here, we extend the knowledge of the protective efficacy of ZK190, a highly potent clone in mice, to NHPs using simple single-plasmid DMAb delivery.

Importantly, the DMAb produces transient expression of anti-ZIKV mAbs *in vivo* that lasts weeks to months in mice, a potential advantage for spacing more frequent biological infusions of mAb that are associated with recombinant mAb delivery. Delivery of a DMAb could potentially span an entire ZIKV infection cycle and could be paired with a long-term prophylactic vaccine to provide rapid protection against ZIKV in at-risk populations ahead of vaccine-induced amnestic immune responses. This would be highly useful during an outbreak. Unlike other gene-encoded approaches, the lack of antivector serology and expression of the DMAb have advantages for the generation of *in vivo* immunity, which could be useful in infection control prior to vaccination being an option. This study demonstrates the feasibility of using nucleic acid for mAb delivery in NHP models, and it supports further development for human translation for multiple disease conditions, including to control infectious diseases like ZIKV.

MATERIALS AND METHODS

Cell Lines and Viruses

HEK293T (American Type Culture Collection [ATCC] CRL-N268, Manassas, VA, USA) and Vero CCL-81 (ATCC CCL-81) cells were maintained in DMEM (Gibco-Invitrogen, Carlsbad, CA, USA) supplemented with 10% fetal bovine serum (FBS, Atlas Biologicals, Fort Collins, CO, USA) and 1% penicillin and streptomycin (Thermo Fisher Scientific). Cell lines were routinely tested to ensure mycoplasma-free culture conditions.

ZIKV strains MR766 (a kind gift from Dr. Susan Weiss) and PR209 (BIOQUAL, MD) were amplified in Vero cells, and stocks were titered by standard plaque assay on Vero cells.

Animals

The 5- to 6-week-old female C57BL/6 (The Jackson Laboratory, Bar Harbor, ME, USA) and 4- to 6-week-old IFNAR^{-}/$^{-}$ (Mutant Mouse Resource and Research Center [MMRRC] repository-The Jackson Laboratory) mice (male and female) were housed and treated in a temperature-controlled, light-cycled facility. All animal protocols were approved by the Institutional Animal Care and Use Committee (IACUC) board (protocol 112761), according to guidelines consistent with the Guide for the Care and Use of Laboratory Animals, 8th edition (the Guide); the Public Health Service Policy on Humane Care and Use of Laboratory Animals (PHS Policy revised 2015); and the Animal Welfare Act and Animal Welfare Regulations (AWRs). All animal research adheres to the standards outlined in OLAW Assurance (A3432-01).

Ten rhesus macaques used in this study were of Chinese origin and acquired by BIOQUAL (Rockville, MD). Animals were housed and cared for at BIOQUAL in accordance with local, state, and federal policies in an Association for Assessment and Accreditation of Laboratory Animal Care International (AAALAC)-accredited facility. All animal experiments were reviewed and approved by the Institutional Animal Care and Use Committee at BIOQUAL. All animals were screened for ZIKV and confirmed seronegative. The animals were divided into two groups based on weight consisting of 2 males and 3 females; the weight of each group averaged 4.98 kg, with a minimum and maximum of 5.30 and 6.00 kg, respectively. The age range of animals was between 4 and 5 years.

Generation of DMAb Constructs

The ZK190 HC and LC families are VH3-30 and VK3-20, respectively.[11] The HC and LC genes for mAb ZK190 and ZK190-LALA were both RNA and DNA sequence optimized. RNA optimization reduces secondary structures and reduces factors negatively impacting expression. Specific DNA mutations in the framework region of ZK190 enhance expression of the DMAb.

mAb ZK190 was isolated from human PBMCs, as previously described.[11,14] ZK190 targets the ZIKV E protein DIII domain. Plasmid DMAb constructs were engineered as previously described.[27,28] Briefly, DMAb constructs encoding fully human IgG1κ mAbs were designed and engineered into a modified-pVax1 mammalian expression vector under the control of a cytomegalovirus immediate-early promoter and bovine growth hormone poly(A) signal. ZK190 mAb antibody sequences were DNA codon optimized (human and mouse) and RNA optimized to minimize secondary structure. A LALA variant, containing mutations at L234A and L235A to abrogate Fc gamma receptor engagement, was also created.[11] The optimized DNA transgenes were then synthesized *de novo* (GenScript, Piscataway, NJ, USA) and inserted into the DMAb expression vector. In a dual-plasmid system, HCs and LCs were expressed from separate plasmids, resulting in three constructs: DMAb-ZK190-HC, DMAb-ZK190-LC, and DMAb-ZK190-LALA-HC. In a single-plasmid system, HCs and LCs are expressed on the same plasmid, resulting in two constructs: DMAb-ZK190 and DMAb-ZK190-LALA.

In Vitro Transfection

At 1 day prior to transfection, HEK293T cells were plated at 0.25×10^6 cells/well in a 12-well tissue culture-treated plate. Cells were transfected with 0.5 μg/DMAb-plasmid using GeneJammer (Agilent Technologies), and cell supernatants were harvested 40 h later. Human IgG DMAb concentration was quantified by ELISA.

Quantitative ELISA

For quantification of total human IgG1κ in cell supernatants, and mouse sera in Figures 1 and S1, 96-well MaxiSorp plates (Nunc) were coated overnight at 4 °C with 10 μg/mL goat anti-human IgG Fc fragment (Bethyl Laboratories). Plates were blocked with 10% FBS in PBS. Sample was diluted in 1× PBS + 0.1% Tween 20 and added to plates for 1 h. A standard curve was generated using purified human IgG1κ (Bethyl Laboratories). Plates were incubated with horseradish peroxidase (HRP)-conjugated goat anti-human kappa light-chain secondary antibody (Bethyl Laboratories) (1:20,000) for 1 h and developed using SigmaFast OPD (Sigma-Aldrich). Absorbance at an optical density (OD) of 450 nm was measured on a Synergy2 plate reader (BioTek Instruments).

Binding and Endpoint Titer ELISA

The 96-well, high-binding immunosorbent plates were coated with 5 μg/mL ZIKV envelope protein (GenScript) and incubated overnight at 4°C. The following day, plates were washed with PBS-T (1% Tween 20) and were incubated in PBS containing 5% non-fat milk and 0.02% Tween 20 for 90 min at 37°C. The plates were washed and incubated with diluted series of samples for 1 h at 37°C. After another wash, the plates were incubated with anti-human IgG (HC + LC [H+L]) conjugated to horseradish peroxidase (SAB3701359, Sigma-Aldrich, St. Louis, MO) in 1:5,000 dilution for 1 h at 37°C. After the final wash, the plates were developed using SigmaFast OPD substrate for 25 min in the dark, then stopped using 2N H_2SO_4. A BioTek Synergy2 plate reader was used to read plates at an OD of 450 nm. Endpoint titers were calculated using the method described in Tebas et al.[9] A sample was considered positive above the cutoff of mean + 3SD.

IM DNA Electroporation

Mice received IM injections of DMAb DNA (50 μg/leg) in the tibialis anterior or quadriceps muscles that had been treated with hyaluronidase (200 U/mL, Sigma-Aldrich, St. Louis, MO), followed by electroporation (IM-EP) using the CELLECTRA 3P adaptive constant current device[29–31] (Inovio Pharmaceuticals, Plymouth Meeting, PA). Serum was collected longitudinally to monitor *in vivo* expression and evaluate binding and neutralization activity. Mice develop an immune response to human antibodies; therefore, to observe long-term kinetics of DMAbs, sera were collected from mice transiently depleted of CD4+ and CD8+ T cells.

Rhesus macaques received 3 sequential administrations of DMAb-DNA (6 mg/administration) on days 0, 3, and 6 by IM injection to the quadriceps muscle, followed by IM-EP using the CELLECTRA 5P adaptive constant current device (Inovio Pharmaceuticals). Serum was collected longitudinally to monitor *in vivo* DMAb pharmacokinetics.

Western Blot

Sample lanes on a NuPAGE 4%–12% Bris-Tris gel (Thermo Fisher Scientific) were loaded with 200 ng ZIKV E protein (GenScript) that was reduced with NuPAGE Sample Reducing Agent (10×) (Thermo Fisher Scientific) for 10 min at 70°C. SeeBlue Pre-stained Protein Standard (Thermo Fisher Scientific) was used as a standard marker. After gel electrophoresis, the samples were transferred to polyvinylidene fluoride (PVDF) membrane Immobilon-FL (IPFL07810, EMD Millipore, MA) using an iBlot 2 system (Thermo Fisher Scientific). The membrane was blocked in OBB (Odyssey Blocking Buffer in PBS, LI-COR Biosciences, NE) for 1 h on a shaker. Sera from individual, DMAb-administered mice were used to probe the membrane (13 ng/mL DMAb-ZK190 or DMAb-ZK190-LALA), diluted in OBB containing 0.1% Tween 20 in 1:2,000 dilution. After 1 h of incubation, the membrane was washed with PBS-T. The membrane was supplied with goat anti-Mouse IRDye 680RD (LI-COR Biosciences, NE) in OBB containing 0.1% Tween 20 and 0.01% SDS in 1:25,000 dilution and was incubated in the dark for 1 h on a shaker. The membrane was washed three times and was scanned using Odyssey CLx Imager (LI-COR Biosciences, NE).

Microneutralization Assay

Sera samples were heat inactivated at 56°C for 30 min before starting the assay. Sera samples were initially diluted 10-fold and then serially diluted 2-fold eight times, all in serum-free DMEM. To each sera dilution, 100 PFU ZIKV strain PR209 in equal volume (50 μL) serum-free DMEM was added, and samples were kept at 37°C for 1.5 h. Flat-bottom 96-well plates with 2.00 × 10^4 Vero cells/well were washed two times with 1× PBS before sera-virus samples were added to wells, and plates were kept at 37°C for 1.5-h incubation. An equal volume (100 μL) of complete DMEM (with 10% FBS, 1% penicillin-streptomycin, and 1% L-glutamine) was added to wells, and plates were returned to the 37°C incubator. After 4 days of incubation, cells were fixed with 4% formaldehyde solution for 45 min, followed by three washes with 1× PBS containing 0.1% v/v Triton X-100. Cells were incubated with mouse anti-flavivirus group antigen mAb (EMD Millipore, MAB10216) for 1.5 h, washed three times with 1× PBS containing 0.1% v/v Tween 20, incubated with a cocktail of IRDye 800CW-anti-mouse IgG secondary antibody (LI-COR Biosciences) + CellTag 700 (LI-COR Biosciences) for 1 h, washed three times with 1× PBS containing 0.1% v/v Tween 20, and left to dry overnight in dark.

Plates were scanned on a LI-COR Odyssey CLx scanner, and the ratio of infected cells:total cells in each well was calculated by dividing the value of the 800-nm signal by the value of the 700-nm signal. The neutralization percentage of each sera dilution was calculated by the following equation: 100 × (1 − ((sample 800/700 ratio)/(virus only 800/700 ratio)). The MN_{50} of neutralization by each volunteer sera was calculated by non-linear regression analysis using Prism 7.

Flow Cytometry-Based Microneutralization Assay

Neutralization of ZIKV infection by mAbs was measured using a microneutralization flow cytometry-based assay. Serum samples were heat inactivated before performing the assay. Different dilutions of mAbs were mixed with ZIKV (MOI of 0.35) for 1 h at 37°C and added to 5000 Vero cells/well in 96-well flat-bottom plates. After 4 days, the cells were fixed with 2% formaldehyde, permeabilized in PBS 1% fetal calf serum (FCS) 0.5% saponin, and stained with the mouse mAb

4G2. The cells were incubated with a goat anti-mouse IgG conjugated to Alexa Fluor488 (Jackson ImmunoResearch Laboratories, 115485164) and analyzed by flow cytometry. The neutralization titer is expressed as the percent reduction versus sera dilution. Day 0 (pre-bleed) and day 7 samples from the same mice are included on each graph.

Lethal ZIKV Challenge

The 4- to 6-week-old IFNAR$^{-/-}$ (n = 8, 4 males and 4 females/group) mice received DMAb-ZK190 (200 μg DMAb-DNA), DMAb-ZK190-LALA (200 μg DMAb-DNA), or an irrelevant control DMAb vector (200 μg pVax1) via IM injection followed by IM-EP 2 days prior to infection. At 1 day prior to infection, ZK190 protein IgG mAb (1.0 mg/kg) was administered to a parallel group of mice by i.p. injection. Mice received a bilateral i.p. injection of low-dose (10^5 PFU) or high-dose (10^6 PFU) ZIKV(PR-209).[32] A control group received no viral infection.

All mice were monitored twice daily for weight loss and survival for 20 days. The percent change in weight was calculated based on the pre-infection weight. Animals were euthanized if they succumbed to hind limb paralysis or lost $\geq$25% of their total weight. Independent from weight loss, mice were also euthanized upon complete paralysis of their hind limbs. Blood was collected 2 days post-infection to assess the amount of human IgG in the serum.

The 4- to 5-year-old male and female rhesus macaques received DMAb-ZK190 (n = 5/group) via IM injection followed by IM-EP 10, 7, and 4 days prior to infection or were naive controls (n = 5). Macaques were challenged at BIOQUAL with 10^4 PFU ZIKV strain PRVABC59.

Viral Loads

RNA was extracted from sera and tissues utilizing the QIAGEN RNeasy kit and RNA stocks were prepared at 1 ng/μL. One-step qRT-PCR was performed using the FastPROBE 1-step qRT-PCR Lo-ROX Kit (Tonbo Biosciences, San Diego, CA). Each reaction was set up according to the manufacturer's protocol and run on an Applied Biosystems Fast 7500 Real-Time PCR instrument (Thermo Fisher Scientific). Serial dilutions of ZIKV RNA (ATCC VR-3252SD, stock 1.2×10^6 genome copies/μL) were prepared to generate a standard curve. The following primer and probe sets were used: forward primer 5′-CCGCTGCCCAACACAAG-3′, reverse primer 5′-CCAC TAACGTTCTTTTGCAGACAT-3′, and probe 5′-FAM-AGCCTAC CTTGACAAGCAGTCAGACACTCAA-BHQ1-3′.[33] The following cycling conditions were used: 1 cycle × 45°C for 10 min, 1 cycle × 95°C for 2 min, and 40 cycles × 95°C for 5 s + 60°C for 30 s. The lower limit of detection of the assay was 12 genome copies. All viral loads are reported per nanogram of total RNA.

Histopathology Analysis

Formalin-fixed, paraffin-embedded spleen, testes, or ovary tissue was sectioned into 5-μm-thick sagittal sections, placed on Superfrost microscope slides (Thermo Fisher Scientific, Hampton, NH, USA), and baked at 37 °C overnight. The sections were de-paraffinized using two changes of xylene and rehydrated by immersing in 100%, 90%, and then 70% ethanol. The sections were stained for nuclear structures using Harris hematoxylin (Surgipath, Buffalo Grove, IL, USA) for 2 min, followed by differentiation in 1% acid alcohol (Surgipath) and treatment with Scott's tap water for 2 min. Subsequently, the sections were counterstained for cytoplasmic structures using eosin (Surgipath) for 2 min. The slides were dehydrated with 70%, 90%, and 100% ethanol; cleared in xylene; and mounted using Permount (Thermo Fisher Scientific). Slides were imaged on a Nikon80i Upright microscope using NIS Elements BR software. Testis and ovary tissue histopathology was evaluated by the PennVet Comparative Pathology Core. This evaluation was performed in a blinded fashion.

Analyses and Statistics

Standard curves and graphs were prepared using GraphPad Prism 6/7. EC_{50} and IC_{50} values were calculated using a non-linear regression of log (reciprocal serum dilution) versus response. Sample size calculations for two independent proportions were calculated with alpha 0.05 and power 0.90. A minimum of n = 5 mice was calculated to be needed in order to ensure adequate power. Survival data were expressed using Kaplan-Meier survival curves with p values calculated by log rank (Mantel-Cox) test. Viral load was analyzed using one-way ANOVA with multiple comparisons. Data were considered significant when $p < 0.05$. The lines in all graphs represent the mean value and error bars represent the SD. No samples or animals were excluded from the analysis. Randomization was not performed for the animal studies. Samples, excluding those sent to the histopathology core for analysis, and animals were not blinded before performing each experiment. Data that support the findings of this study are available from the corresponding author upon reasonable request.

SUPPLEMENTAL INFORMATION

Supplemental Information can be found online at https://doi.org/10.1016/j.ymthe.2019.03.005.

AUTHOR CONTRIBUTIONS

A.P. and R.N.E. designed and performed experiments, analyzed data, and wrote the manuscript. S.B.K. performed experiments, analyzed data, and wrote the manuscript. D.H.P. performed experiments and wrote the manuscript. M.B., S.S., K.A., P.B., K.S., M.B., H.C., and J.W.A. performed experiments. A.C.D., S.R., J.C., J.Y., T.R.F.S., K.B., G.G. participated in data analysis. K.M., D.C., L.H., and D.B.W. provided administrative, technical, or supervisory support.

CONFLICTS OF INTEREST

K.M. received grants from DARPA and Inovio, consulting fees from Inovio related to DNA vaccine development, and has a pending patent application (to Inovio) for the delivery of DNA-encoded monoclonal antibodies. J.W.A., J.M., S.R., J.C., J.Y., T.R.F.S., K.B., G.G., and L.H. are employees of Inovio Pharmaceuticals and as such receive salary and benefits, including ownership of stock and stock options,

from the company. K.S., M.B., and D.C. are employees of Humabs BioMed: a subsidiary of VirBiotechnology. D.B.W. has received grant funding, participates in industry collaborations, has received speaking honoraria, and has received fees for consulting, including serving on scientific review committees and board services. Remuneration received by D.B.W. includes direct payments or stock or stock options, and, in the interest of disclosure, he notes potential conflicts associated with this work with Inovio and possibly others. In addition, he has a patent DNA vaccine delivery pending to Inovio. All other authors declare no competing interests.

ACKNOWLEDGMENTS

We kindly thank the Wistar Histotechnology Core and The PennVet Comparative Pathology Core for their services. This work was supported in part by the Bill & Melinda Gates Foundation (OPP1163327). R.N.E. was supported by the Emerging Infectious Disease NIH Training Grant (T32-AI055400).

REFERENCES

1. Shankar, A., Patil, A.A., and Skariyachan, S. (2017). Recent Perspectives on Genome, Transmission, Clinical Manifestation, Diagnosis, Therapeutic Strategies, Vaccine Developments, and Challenges of Zika Virus Research. Front. Microbiol. *8*, 1761.
2. Nicastri, E., Castilletti, C., Liuzzi, G., Iannetta, M., Capobianchi, M.R., and Ippolito, G. (2016). Persistent detection of Zika virus RNA in semen for six months after symptom onset in a traveller returning from Haiti to Italy, February 2016. Euro Surveill. *21*, 30314.
3. Barzon, L., Pacenti, M., Franchin, E., Lavezzo, E., Trevisan, M., Sgarabotto, D., and Palù, G. (2016). Infection dynamics in a traveller with persistent shedding of Zika virus RNA in semen for six months after returning from Haiti to Italy, January 2016. Euro Surveill. *21*, 30316.
4. van den Pol, A.N., Mao, G., Yang, Y., Ornaghi, S., and Davis, J.N. (2017). Zika Virus Targeting in the Developing Brain. J. Neurosci. *37*, 2161–2175.
5. Huang, W.-C., Abraham, R., Shim, B.-S., Choe, H., and Page, D.T. (2016). Zika virus infection during the period of maximal brain growth causes microcephaly and corticospinal neuron apoptosis in wild type mice. Sci. Rep. *6*, 34793.
6. Musso, D., Roche, C., Robin, E., Nhan, T., Teissier, A., and Cao-Lormeau, V.-M. (2015). Potential sexual transmission of Zika virus. Emerg. Infect. Dis. *21*, 359–361.
7. Ma, W., Li, S., Ma, S., Jia, L., Zhang, F., Zhang, Y., Zhang, J., Wong, G., Zhang, S., Lu, X., et al. (2016). Zika Virus Causes Testis Damage and Leads to Male Infertility in Mice. Cell *167*, 1511–1524.e10.
8. Griffin, B.D., Muthumani, K., Warner, B.M., Majer, A., Hagan, M., Audet, J., Stein, D.R., Ranadheera, C., Racine, T., De La Vega, M.A., et al. (2017). DNA vaccination protects mice against Zika virus-induced damage to the testes. Nat. Commun. *8*, 15743.
9. Tebas, P., Roberts, C.C., Muthumani, K., Reuschel, E.L., Kudchodkar, S.B., Zaidi, F.I., White, S., Khan, A.S., Racine, T., Choi, H., et al. (2017). Safety and Immunogenicity of an Anti-Zika Virus DNA Vaccine—Preliminary Report. N. Engl. J. Med. Published online October 4, 2017. https://doi.org/10.1056/NEJMoa1708120.
10. Sapparapu, G., Fernandez, E., Kose, N., Cao, B., Fox, J.M., Bombardi, R.G., Zhao, H., Nelson, C.A., Bryan, A.L., Barnes, T., et al. (2016). Neutralizing human antibodies prevent Zika virus replication and fetal disease in mice. Nature *540*, 443–447.
11. Stettler, K., Beltramello, M., Espinosa, D.A., Graham, V., Cassotta, A., Bianchi, S., Vanzetta, F., Minola, A., Jaconi, S., Mele, F., et al. (2016). Specificity, cross-reactivity, and function of antibodies elicited by Zika virus infection. Science *353*, 823–826.
12. Magnani, D.M., Rogers, T.F., Beutler, N., Ricciardi, M.J., Bailey, V.K., Gonzalez-Nieto, L., Briney, B., Sok, D., Le, K., Strubel, A., et al. (2017). Neutralizing human monoclonal antibodies prevent Zika virus infection in macaques. Sci. Transl. Med. *9*, eaan8184.
13. Robbiani, D.F., Bozzacco, L., Keeffe, J.R., Khouri, R., Olsen, P.C., Gazumyan, A., Schaefer-Babajew, D., Avila-Rios, S., Nogueira, L., Patel, R., et al. (2017). Recurrent Potent Human Neutralizing Antibodies to Zika Virus in Brazil and Mexico. Cell *169*, 597–609.e11.
14. Wang, J., Bardelli, M., Espinosa, D.A., Pedotti, M., Ng, T.-S., Bianchi, S., Simonelli, L., Lim, E.X.Y., Foglierini, M., Zatta, F., et al. (2017). A Human Bi-specific Antibody against Zika Virus with High Therapeutic Potential. Cell *171*, 229–241.e15.
15. Muthumani, K., Griffin, B.D., Agarwal, S., Kudchodkar, S.B., Reuschel, E.L., Choi, H., Kraynyak, K.A., Duperret, E.K., Keaton, A.A., Chung, C., et al. (2016). *In vivo* protection against ZIKV infection and pathogenesis through passive antibody transfer and active immunisation with a prMEnv DNA vaccine. NPJ Vaccines *1*, 16021.
16. Joguet, G., Mansuy, J.-M., Matusali, G., Hamdi, S., Walschaerts, M., Pavili, L., Guyomard, S., Prisant, N., Lamarre, P., Dejucq-Rainsford, N., et al. (2017). Effect of acute Zika virus infection on sperm and virus clearance in body fluids: a prospective observational study. Lancet Infect. Dis. *17*, 1200–1208.
17. Govero, J., Esakky, P., Scheaffer, S.M., Fernandez, E., Drury, A., Platt, D.J., Gorman, M.J., Richner, J.M., Caine, E.A., Salazar, V., et al. (2016). Zika virus infection damages the testes in mice. Nature *540*, 438–442.
18. Nugent, E.K., Nugent, A.K., Nugent, R., and Nugent, K. (2017). Zika Virus: Epidemiology, Pathogenesis and Human Disease. Am. J. Med. Sci. *353*, 466–473.
19. Sharp, T.M., Tomashek, K.M., Read, J.S., Margolis, H.S., and Waterman, S.H. (2017). A New Look at an Old Disease: Recent Insights into the Global Epidemiology of Dengue. Curr. Epidemiol. Rep. *4*, 11–21.
20. Beasley, D.W.C., McAuley, A.J., and Bente, D.A. (2015). Yellow fever virus: genetic and phenotypic diversity and implications for detection, prevention and therapy. Antiviral Res. *115*, 48–70.
21. Wiley, C.A., and Chimelli, L. (2017). Human Zika and West Nile virus neurological infections: What is the difference? Neuropathology *37*, 393–397.
22. Shi, W., Zhang, Z., Ling, C., Carr, M.J., Tong, Y., and Gao, G.F. (2016). Increasing genetic diversity of Zika virus in the Latin American outbreak. Emerg. Microbes Infect. *5*, e68.
23. Pantoja, P., Pérez-Guzmán, E.X., Rodríguez, I.V., White, L.J., González, O., Serrano, C., Giavedoni, L., Hodara, V., Cruz, L., Arana, T., et al. (2017). Zika virus pathogenesis in rhesus macaques is unaffected by pre-existing immunity to dengue virus. Nat. Commun. *8*, 15674.
24. Terzian, A.C.B., Schanoski, A.S., Mota, M.T.O., da Silva, R.A., Estofolete, C.F., Colombo, T.E., Rahal, P., Hanley, K.A., Vasilakis, N., Kalil, J., and Nogueira, M.L. (2017). Viral Load and Cytokine Response Profile Does Not Support Antibody-Dependent Enhancement in Dengue-Primed Zika Virus-Infected Patients. Clin. Infect. Dis. *65*, 1260–1265.
25. Miner, J.J., and Diamond, M.S. (2017). Dengue Antibodies, then Zika: A Fatal Sequence in Mice. Immunity *46*, 771–773.
26. van Meer, P.J.K., Kooijman, M., Brinks, V., Gispen-de Wied, C.C., Silva-Lima, B., Moors, E.H.M., and Schellekens, H. (2013). Immunogenicity of mAbs in non-human primates during nonclinical safety assessment. MAbs *5*, 810–816.
27. Muthumani, K., Block, P., Flingai, S., Muruganantham, N., Chaaithanya, I.K., Tingey, C., Wise, M., Reuschel, E.L., Chung, C., Muthumani, A., et al. (2016). Rapid and Long-Term Immunity Elicited by DNA-Encoded Antibody Prophylaxis and DNA Vaccination Against Chikungunya Virus. J. Infect. Dis. *214*, 369–378.
28. Flingai, S., Plummer, E.M., Patel, A., Shresta, S., Mendoza, J.M., Broderick, K.E., Sardesai, N.Y., Muthumani, K., and Weiner, D.B. (2015). Protection against dengue disease by synthetic nucleic acid antibody prophylaxis/immunotherapy. Sci. Rep. *5*, 12616.
29. Broderick, K.E., and Humeau, L.M. (2015). Electroporation-enhanced delivery of nucleic acid vaccines. Expert Rev. Vaccines *14*, 195–204.
30. Hirao, L.A., Wu, L., Khan, A.S., Satishchandran, A., Draghia-Akli, R., and Weiner, D.B. (2008). Intradermal/subcutaneous immunization by electroporation improves

plasmid vaccine delivery and potency in pigs and rhesus macaques. Vaccine *26*, 440–448.

31. Amante, D.H., Smith, T.R.F., Mendoza, J.M., Schultheis, K., McCoy, J.R., Khan, A.S., Sardesai, N.Y., and Broderick, K.E. (2015). Skin Transfection Patterns and Expression Kinetics of Electroporation-Enhanced Plasmid Delivery Using the CELLECTRA-3P, a Portable Next-Generation Dermal Electroporation Device. Hum. Gene Ther. Methods *26*, 134–146.

32. Bradley, M.P., and Nagamine, C.M. (2017). Animal Models of Zika Virus. Comp. Med. *67*, 242–252.

33. Lanciotti, R.S., Kosoy, O.L., Laven, J.J., Velez, J.O., Lambert, A.J., Johnson, A.J., Stanfield, S.M., and Duffy, M.R. (2008). Genetic and serologic properties of Zika virus associated with an epidemic, Yap State, Micronesia, 2007. Emerg. Infect. Dis. *14*, 1232–1239.

YMTHE, Volume *27*

Supplemental Information

In Vivo Delivery of a DNA-Encoded Monoclonal Antibody Protects Non-human Primates against Zika Virus

Rianne N. Esquivel, Ami Patel, Sagar B. Kudchodkar, Daniel H. Park, Karin Stettler, Martina Beltramello, Jeffrey W. Allen, Janess Mendoza, Stephanie Ramos, Hyeree Choi, Piyush Borole, Kanika Asija, Mamadou Bah, Shareef Shaheen, Jing Chen, Jian Yan, Amy C. Durham, Trevor R.F. Smith, Kate Broderick, Ghiabe Guibinga, Kar Muthumani, Davide Corti, Laurent Humeau, and David B. Weiner

Supplemental Figures

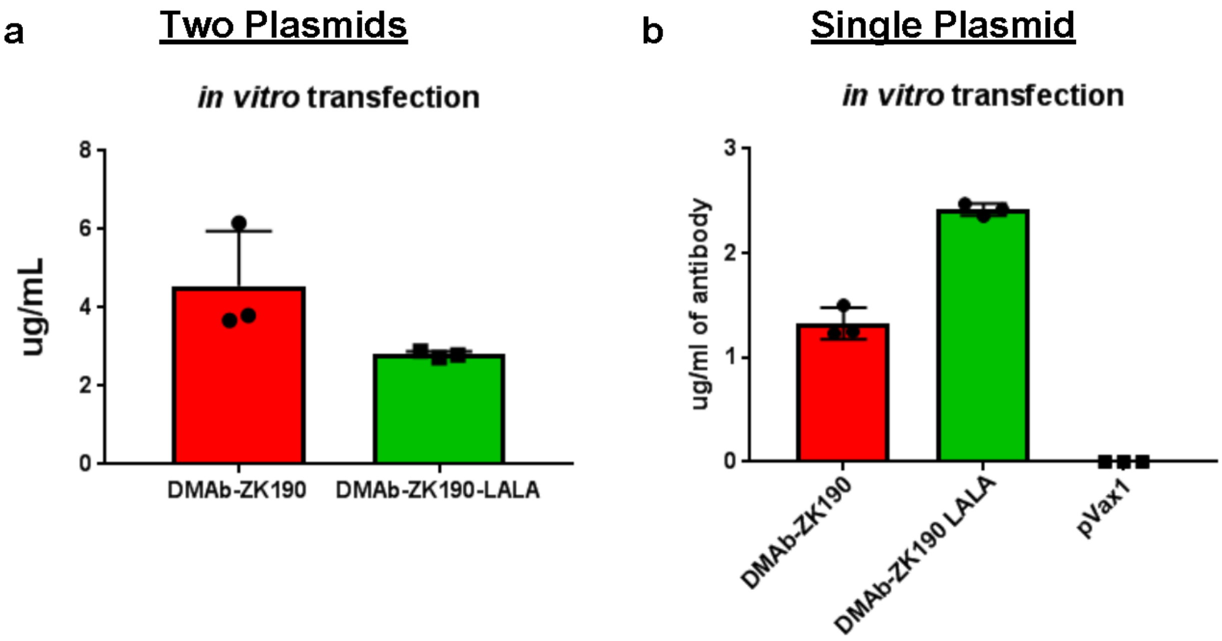

Supplemental Figure 1: **ZIKV DMAb expression in vitro.** In vitro expression of DMAb-ZK190 two plasmid (**a**) (n=3) or one plasmid (**b**) (n=3) system in transfected HEK 293 cells. Cell supernatant was harvested 48 hours post-transfection and human IgG1 expression was detected and quantified by ELISA. For each experiment “n” refers to biological replicates. Error bars refer to standard deviation.

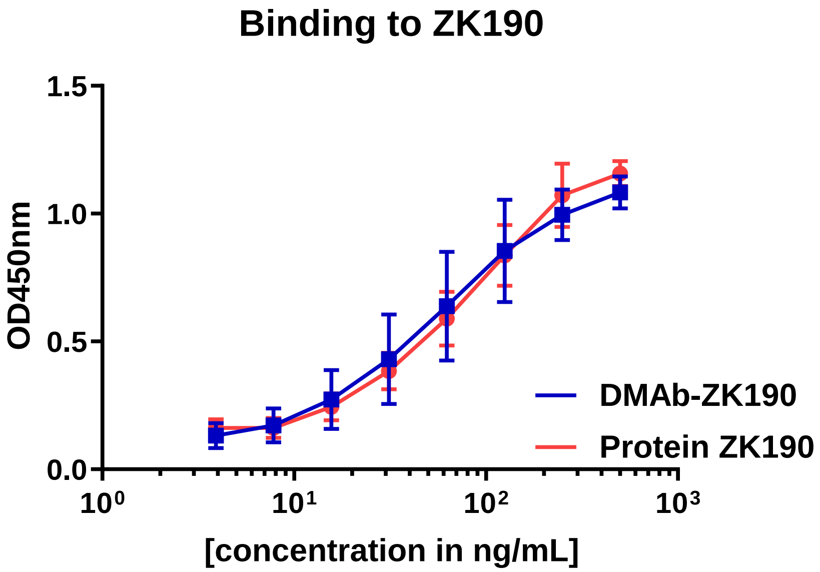

Supplemental Figure 2: **Quantification of ZIKV DMAb-ZK190 binding using recombinant ZK190 protein as a standard.** Sera from ZK190-DMAb injected animals (n=7) was quantified using recombinant ZK190 protein as a standard. Plates were coated with ZIKV E protein and detected as outline in the Binding ELISA methods section.

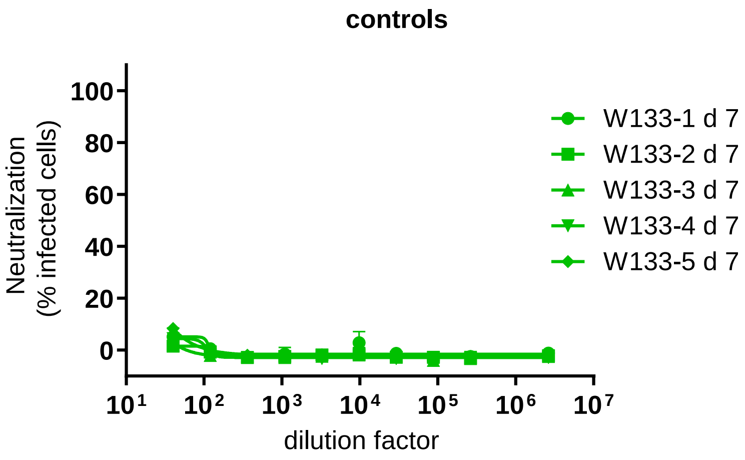

Supplemental Figure 3: *In vitro* neutralization activity of naïve mouse sera in a flow cytometry neutralization assay. Serial dilutions of sera from naïve mice (n=5) were evaluated in vitro in a flow-based assay for their ability to block ZIKV H/PF/2013 (100 p.f.u.) infection of Vero cells. Linear regression analysis was used to determine concentration of sera that neutralized infection by 50% compared to wells receiving virus only. For each experiment "n" refers to biological replicates.

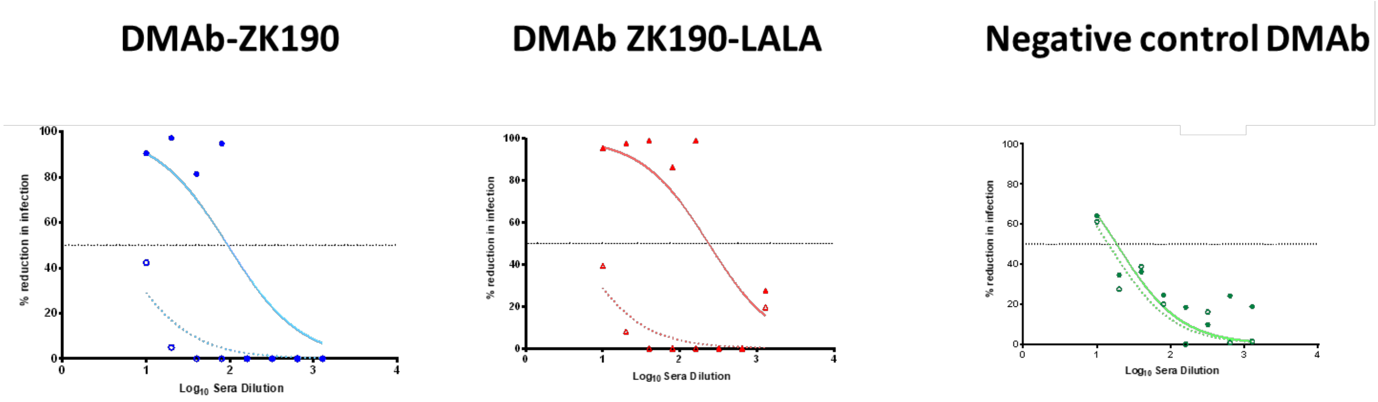

Supplemental Figure 4: *In vitro* neutralization activity of DMAb-ZK190 and DMAb-ZK190 LALA. (a) Serial dilutions of pooled day 7 sera from mice (n=5) injected with DMAb-ZK190, DMAb-ZK190 LALA, or a negative control DMAb were evaluated in an immunostaining-based assay for their ability to block ZIKV PR209 (100 p.f.u.) infection of Vero cells. Linear regression analysis used to determine concentration of DMAb in sera that neutralized infection by 50% (dotted line) compared to wells receiving virus only. For each experiment "n" refers to biological replicates.

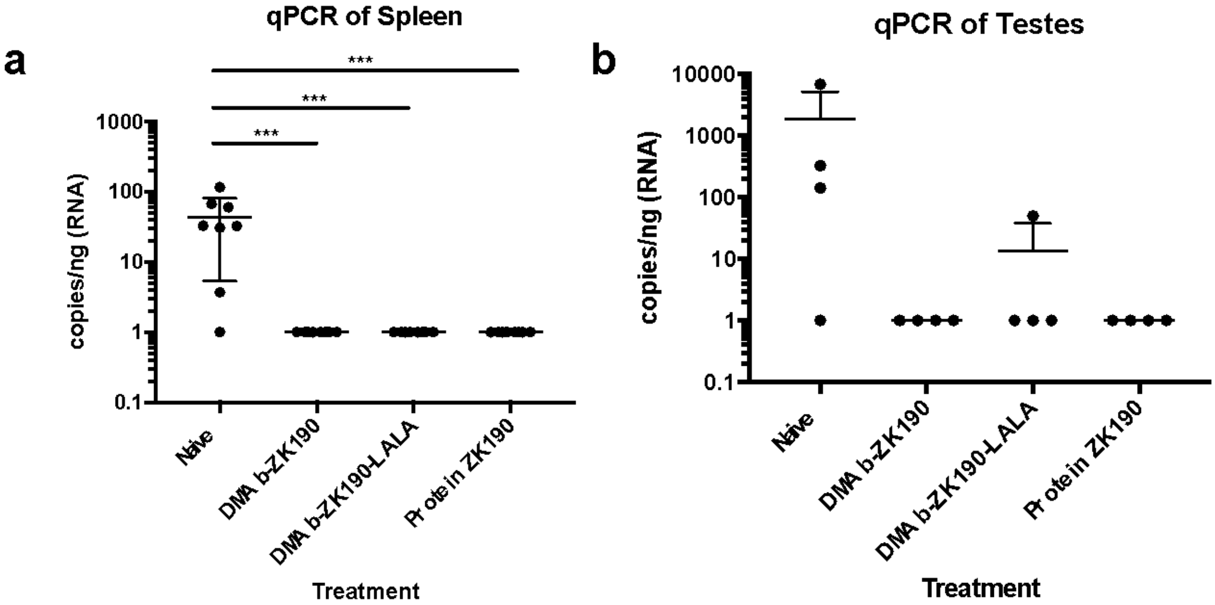

Supplemental Figure 5: Viral load in tissues following high dose ZIKV mouse challenge. Tissues were harvested from DMAb-ZK190, DMAb-ZK190-LALA, protein ZK190, and pVax11 control mice challenged with ZIKV (10^6 PFU dose). RNA was extracted from A) spleen (n=8) and B) testes (n=4) at the terminal endpoint. ZIKV genome copies/ng of RNA were detected by qRT-PCR. For each experiment "n" refers to biological replicates. Error bars refer to standard deviation.

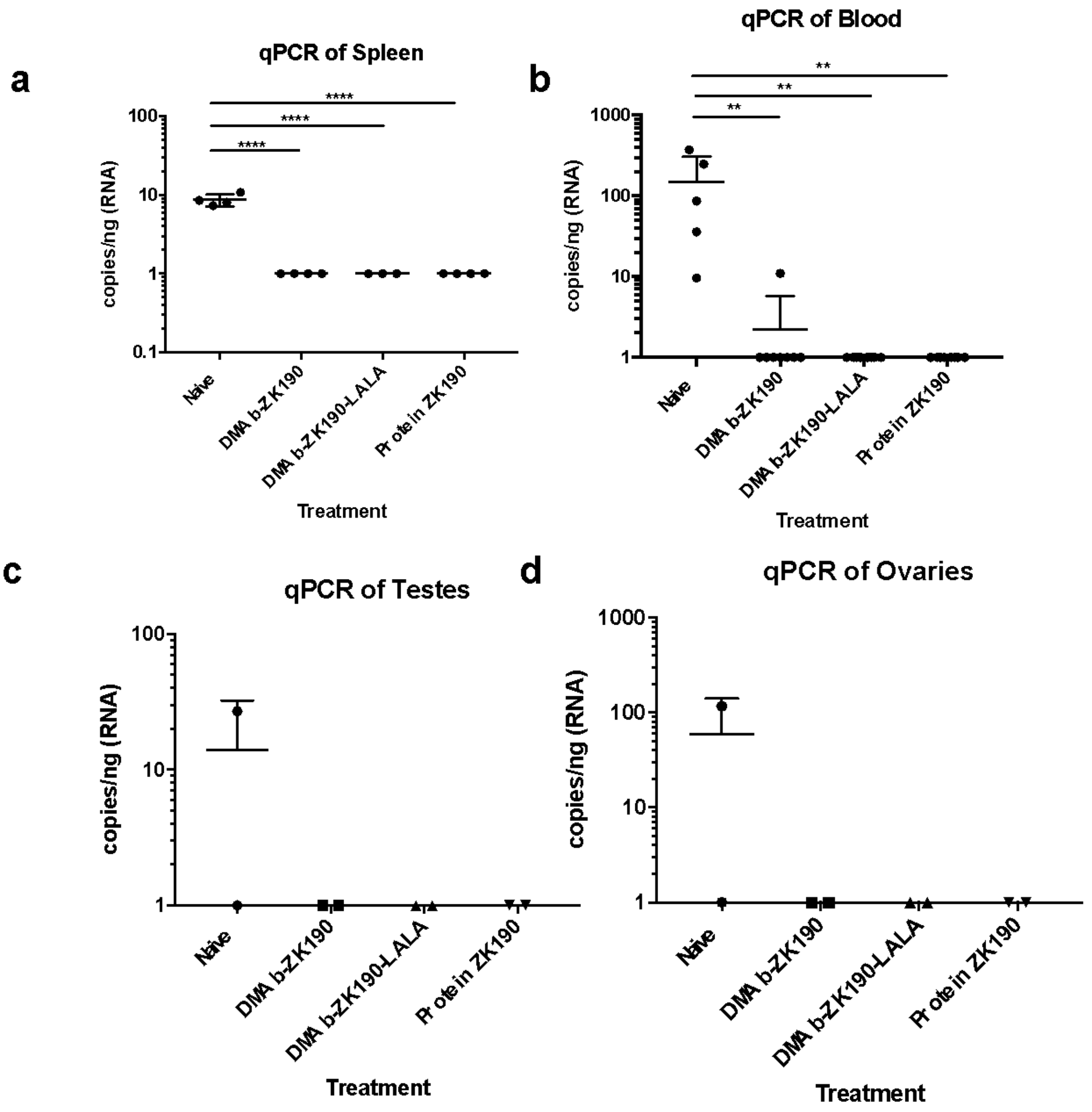

Supplemental Figure 6: Viral load in tissues following low dose ZIKV mouse challenge. Tissues were harvested from DMAb-ZK190, DMAb-ZK190-LALA, protein ZK190, and pVax11 control mice challenged with ZIKV (10^5 PFU dose). RNA was extracted from A) spleen (n=4, DMAb-ZK190, protein ZK190, pVax) (n=3 DMAb-ZK190-LALA), B) ovaries (n=2), C) testes (n=2) and D) blood (n=5, pVax)(n=8, DMAb-ZK190, DMAb-ZK190-LALA, protein ZK190) at

the terminal endpoint. ZIKV genome copies/ng of RNA were detected by qRT-PCR. For each experiment “n” refers to biological replicates. Error bars refer to standard deviation.

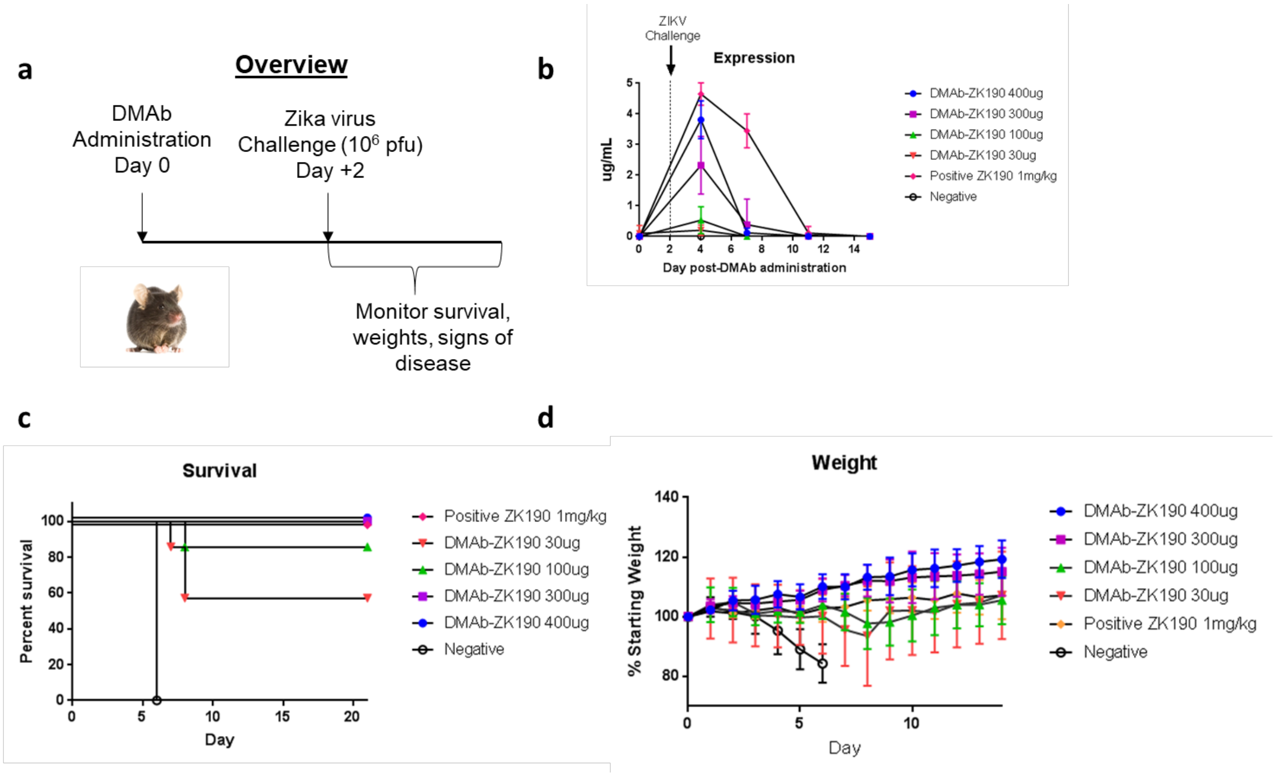

Supplemental Figure 7*: In vivo* protection by single plasmid DMAb-ZK190. **a**) Overview of the injection regimen. DMAbs were administered on day 0 and serum was collected on day 0, 4, 7, and 11 post-lethal challenge with 10^6 PFU of Zika Strain PR209. Animals were monitored for 21 days post-challenge for signs of disease and weight loss. **b**) Serum human IgG levels at day 0, 4, 7, and 11 post challenge. **c**) Survival of 30 ug, 100 ug, 300 ug, or 400 ug ZK190-DMAb receiving mice (n=7) compared to negative control (n=7) and protein IgG (n=7). **d**) Percentage weight change for negative control group receiving DMAb empty vector pVax11 (100 µg/mouse) compared to mice receiving treatment group ZK190 (30ug, 100ug, 300 µg or 400 ug) or protein ZK190 (1 mg/kg) For each experiment “n” refers to biological replicates. Error bars refer to standard deviation.

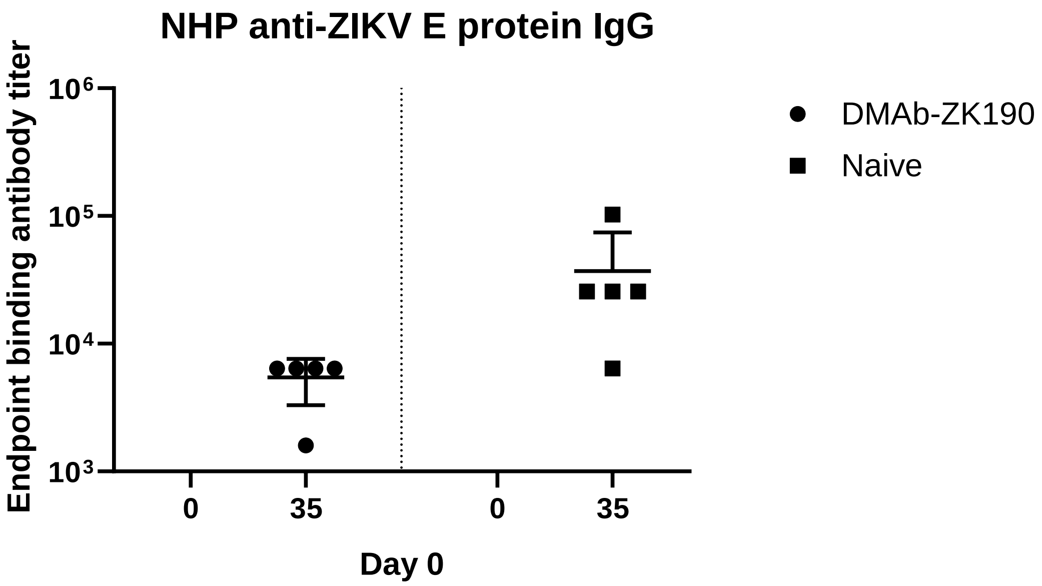

Supplemental Figure 8: Serum ZIKV endpoint titres in DMAb-ZK190 administered and naïve macaques following challenge. Endpoint binding antibody titres of naïve and ZK190-DMAb receiving NHPs at day 35 post-challenge as measured by binding ELISA on plates coated with ZIKV E protein (n=5).

Supplemental Table 1. Histopathology of testes of DMAb, protein or pVax treated mice post- Zika challenge.

Treatment Group	Histopathology
**DMAb-ZK190**	Testicle: Spermatogenesis<br>Epididymis: Maturing spermatids
**DMAb-ZK190-LALA**	Testicle: Spermatogenesis<br>Epididymis: Maturing spermatids
**Protein ZK190**	Testicle: Spermatogenesis<br>Epididymis: Maturing spermatids
**pVax 10^6 pfu**	Testicle: Spermatogenesis<br>Epididymis: Maturing spermatids
**pVax 10^5 pfu**	Testicle: Severe necrotizing and histiocytic orchitis with degeneration and regional parenchymal loss and edema (~40%)<br>Epididymis: Reduced sperm; multifocal luminal cell debris

Supplemental Table 2. Histopathology of ovaries of DMAb, protein or pVax treated mice post- Zika challenge.

Treatment Group	Histopathology
**DMAb-ZK190**	Uterus (myometrium): Mild granulocytic infiltrate<br>Ovary: multiple corpora lutea; folliculogenesis
**DMAb-ZK190-LALA**	Uterus (myometrium): Mild granulocytic infiltrate<br>Ovary: multiple corpora lutea; folliculogenesis
**Protein ZK190**	Uterus (myometrium): Mild granulocytic infiltrate<br>Ovary: multiple corpora lutea; folliculogenesis
**pVax 10^6 pfu**	Uterus (myometrium): Mild granulocytic infiltrate<br>Ovary: multiple corpora lutea; folliculogenesis
**pVax 10^5 pfu**	Mild glandular dilation with luminal debris; mild granulocytic infiltrate
